# Testosterone Regulates Sex-Dependent Immune Responses and Androgen Receptor Expression During *Plasmodium berghei* ANKA Infection

**DOI:** 10.3390/ijms27198649

**Published:** 2026-09-27

**Authors:** Teresita de Jesús Nolasco-Pérez, Luis Antonio Cervantes-Candelas, Víctor Hugo Salazar-Castañón, Fidel Orlando Buendía-González, David Roberto Chavira-Ramírez, Martha Legorreta-Herrera

**Affiliations:** 1 Laboratorio de Inmunología Molecular, Unidad de Investigación Química Computacional, Síntesis y Farmacología de Moléculas de Interés Biológico, División de Estudios de Posgrado e Investigación, Facultad de Estudios Superiores Zaragoza, Universidad Nacional Autónoma de México (UNAM), Iztapalapa, Ciudad de México 09230, Mexico; teresyta.qfb@gmail.com (T.d.J.N.-P.); luis.cervantes@zaragoza.unam.mx (L.A.C.-C.); mestilom@hotmail.com (V.H.S.-C.); fidelbuendia2@gmail.com (F.O.B.-G.); 2 Posgrado en Ciencias Biológicas, Universidad Nacional Autónoma de México (UNAM), Unidad de Posgrado, Edificio D, 1° Piso, Circuito de Posgrados, Ciudad Universitaria, Coyoacán, Ciudad de México 04510, Mexico; 3 Departamento de Biología de la Reproducción, Instituto Nacional de Ciencias Médicas y Nutrición Salvador Zubirán, Ciudad de México 14080, Mexico; roberto.chavira@incmnsz.mx

**Keywords:** testosterone levels, letrozole, androgen receptor, *Plasmodium berghei*, sexual dimorphism

## Abstract

Malaria exhibits pronounced sexual dimorphism, with males experiencing greater disease severity and mortality than females do. Although these differences have been partially attributed to the immunosuppressive effects of testosterone, the underlying mechanisms remain poorly understood. Here, we investigated the role of testosterone in sex-specific immune responses by administering a combination of letrozole and testosterone (LT) to male and female mice infected with *Plasmodium berghei* ANKA. LT treatment increased testosterone concentrations in both sexes and was associated with higher parasitemia, and splenic index mainly in males, whereas hemoglobin levels decreased only in males. In females, LT increased macrophage numbers, reduced B-cell numbers, enhanced IgM production, increased androgen receptor (*AR*) expression, and decreased estrogen receptor-α (*ERα*) expression. In contrast, LT selectively reduced IL-17A levels in males without modifying *AR* expression. In addition, the IL-2, IFN-γ, and IL-10 levels remained unchanged in both sexes. These findings demonstrate that testosterone differentially regulates immune responses during malaria through sex-specific modulation of steroid hormone receptor expression. This study provides insight into how testosterone contributes to sex-dependent immune responses to malaria. In turn, elucidating the role of testosterone in these differences may help identify new therapeutic opportunities tailored to biological sex and ultimately contribute to the development of more effective strategies for managing infectious diseases.

## 1. Introduction

Malaria is the most significant parasitic disease; it caused 610,000 deaths in 2024 [1] and is sexually dimorphic, with men experiencing higher mortality rates and more severe symptoms than women do [2]. These differences have been attributed to the immunomodulatory effects of sex hormones, in particular, testosterone and estrogens, which regulate various aspects of innate and adaptive immune responses [3,4]. Testosterone has been associated with immunosuppressive effects [4]; testosterone affects spleen cell populations and cytokine synthesis and has been linked to oxidative damage in mice [5]. Immune cells in both males and females possess testosterone receptors; however, the hormone modulates the immune response differently in the two sexes [3,6].

Although sexual dimorphism in malaria has been recognized for decades, the causes of differences in severity and mortality are completely unknown; therefore, mouse models using different strains are employed to simulate the lethality of *Plasmodium falciparum* infections, as is the case with the CBA/Ca strain. In various studies, the concentration of steroid hormones is modified to elucidate the effect of these hormones on the dimorphic pattern of symptom severity and mortality; the main findings regarding the role of testosterone in this dimorphic response have been obtained primarily in males using strategies such as gonadectomy or hormone administration [7,8]. The main disadvantage of gonadectomy is that it eliminates all the hormones produced in the gonads, whereas increasing testosterone concentration promotes its conversion to estrogens by aromatase, making it difficult to determine specifically the contribution of testosterone to sexual dimorphism in malaria. To overcome this limitation and determine only the effect of testosterone, we first administered testosterone in combination with letrozole, a potent aromatase inhibitor, to block its conversion to estrogens, and found that the LT combination increased the hormone concentration. Furthermore, it increased the populations of CD19^+^ and CD8^+^ cells while decreasing the number of MAC-3^+^ cells in a hormone concentration-dependent manner. Furthermore, the LT combination increases the concentrations of IL-4 and TNF-α and decreases those of IL-17, altering the inflammatory response, increasing parasitemia and exacerbating anemia. However, these findings have been documented only in males [9].

Significant progress has been made in understanding the role of testosterone in malaria. However, the mechanisms by which this hormone modulates the anti-malaria immune response in a dimorphic manner have not yet been fully elucidated. Furthermore, whether the expression of androgen and estrogen receptors is modulated by testosterone concentration and sex during *Plasmodium* infection is unknown. Similarly, the functional consequences of this regulation in both sexes remain unknown. Consequently, in this study, we administered a combination of LT to female or male mice infected with *Plasmodium berghei* ANKA (*P. berghei* ANKA) and investigated the role of testosterone in the sexual dimorphism of the immune response. We found that, rather than being immunosuppressive, testosterone modulates immune cell populations in the spleen and proinflammatory cytokines in a sex-dependent manner; furthermore, infection in males suppressed the expression of androgen receptor (*AR*) and estrogen receptor α (*ERα*) regardless of the testosterone concentration. In contrast, in infected females, increasing the testosterone concentration increased *AR* expression and reduced *ERα* expression; the implications of these findings for the dimorphism of the immune response in malaria are discussed.

## 2. Results

### 2.1. Administration of LT Increased Free Testosterone Levels in Both Sexes

To assess the role of testosterone in the sexual dimorphism of the immune response in mice infected with *P. berghei* ANKA, the testosterone concentration was increased, and testosterone was administered simultaneously with letrozole (LT); letrozole is a selective aromatase inhibitor that prevents the conversion of testosterone to estrogens [10]. As this treatment could also alter the concentrations of 17β-estradiol, dehydroepiandrosterone (DHEA), and progesterone, these steroid hormones were quantified. In uninfected females, neither letrozole nor testosterone had a statistically significant effect on free testosterone levels; however, infection reduced the concentration of this hormone. Furthermore, infected females treated with testosterone or LT showed a significant increase in the free testosterone concentration. In males, the administration of letrozole or testosterone significantly increased the levels of this hormone; the effect was even greater with the LT combination, regardless of infection status, thereby eliminating the dimorphic patterns observed between the two sexes under our experimental conditions (Figure 1A). The testosterone concentrations in the untreated, vehicle-treated, and LT-treated groups are presented in Supplementary Figure S1.

With respect to 17β-estradiol, in females, testosterone and letrozole did not significantly alter the concentration of this hormone, regardless of infection status. In contrast, compared with mice that received vehicle or letrozole, males that received LT showed a reduction in 17β-estradiol (Figure 1B).

Furthermore, infection with *P. berghei* ANKA eliminated the sexually dimorphic pattern observed in uninfected mice; infected females increased their DHEA concentration to match that of males under the same conditions, in which the DHEA concentration remained constant regardless of infection or the increase in testosterone concentration (Figure 1C).

With respect to the effects of increased testosterone on progesterone levels, compared with uninfected males, uninfected females had higher levels. Interestingly, infection significantly reduced progesterone levels in females, eliminating the sexually dimorphic pattern observed in uninfected mice (Figure 1D).

### 2.2. Testosterone Modulated AR Expression in the Spleen in a Dimorphic Manner

To determine whether the concentration-dependent immunological effects of testosterone were associated with changes in *AR* expression, given that testosterone activates the classical and nongenomic pathways via the *AR* in response to different stimuli, we assessed whether *AR* expression is modulated in a dimorphic manner in the spleens and brains of mice infected with *P. berghei* ANKA. We assessed *AR* expression in the spleens and brains of males and females. Compared with vehicle, infection significantly reduced *AR* expression in males treated with letrozole; in contrast, *AR* expression increased in infected females treated with LT, suggesting a link to the increase in testosterone concentration (Figure 2B). In addition, compared with vehicle or letrozole treatments, testosterone treatment significantly increased *AR* expression in the brains of infected females (Figure 2C).

In contrast, the expression of *ERα* in the spleen significantly increased in females infected with *P. berghei* ANKA, suggesting that the low expression of this receptor is related to the increase in testosterone concentration (Figure 2D). Furthermore, under the same conditions, only infected males showed a statistically significant increase in *ERα* expression in the brain following testosterone administration (Figure 2E).

### 2.3. An Increase in Testosterone Levels Had a Dimorphic Effect on Parasitemia, Hemoglobin Concentration, Body Weight, and Temperature

Having demonstrated that infection with *P. berghei* ANKA reduced free testosterone levels in females and that treatment with the LT combination increased free testosterone levels in both sexes and that this modified the expression of androgen receptors in the spleen and brain after LT treatment, without altering those of 17β-estradiol, DHEA and progesterone, we wondered whether the increase in testosterone altered the course of infection in males and females because testosterone concentrations increased.

As the spleen is the main organ responsible for the clearance of parasitized erythrocytes [11] and the inflammatory response induced by infection promotes the development of splenomegaly [12], in addition to the fact that androgens modulate the immune response in organs such as the spleen and the brain [3,9], we assessed the effects of increased testosterone concentration on parasitemia, hemoglobin concentration, body weight, body temperature, and the splenic index as indirect indicators of the progression of infection and the host response.

A markedly sexually dimorphic pattern was observed for parasitemia on days 7 and 8 PI; the increase in testosterone concentration mainly affected males, who developed significantly higher parasitemia levels than females did, particularly following treatment with LT (*p* values < 0.0001) (Figure 3A). Consistently, treatment with testosterone or LT significantly reduced the hemoglobin concentration in males. In contrast, females treated with LT maintained hemoglobin concentrations similar to those of their respective controls, demonstrating a clear sexually dimorphic pattern with statistical support in response to increased testosterone levels (Figure 3B).

In addition, infected males had significantly reduced body weight in all infected groups compared with their respective uninfected controls. Treatment with LT did not significantly alter body weight in infected males. Interestingly, infected females exhibited a significant increase in body weight compared with uninfected females except for the group treated with testosterone or LT (Figure 3C).

With respect to body temperature, no statistically significant changes were observed in uninfected males under the experimental conditions evaluated. In contrast, infection significantly decreased body temperature in females treated with vehicle. Treatment with letrozole or testosterone significantly increased body temperature in infected females compared to the vehicle-treated group. These results demonstrated a sexually dimorphic pattern in the body temperature response to modulation of testosterone concentration (Figure 3D).

In addition, infection with *P. berghei* ANKA increased the splenic index in all groups of female and male mice, which generated a dimorphic pattern in the groups treated with L, T and LT (Figure 3E). On the basis of these results, we assessed whether increased testosterone modulated the main cellular populations of the immune system in the spleen in a sexually dimorphic manner. We also quantified the concentrations of cytokines and antibodies associated with the immune response and with the development of severe malaria [7,13,14].

### 2.4. The Concentrations of IFN-γ and IL-2 Decreased in a Testosterone-Concentration-Dependent Manner in Females

Given that the increase in testosterone induced sexually dimorphic patterns in the mRNA expression of androgen and estrogen receptors, parasitemia and hemoglobin concentration and that infection with *P. berghei* ANKA modifies the composition of immune cell populations [15], we assessed whether these differences were due to testosterone altering the number of cell populations in the spleen. First, we analyzed the effector cell populations with cytotoxic activity involved in controlling infection, particularly NK cells and lymphocytes (CD3^+^CD8^+^) [16].

We found that compared with females, uninfected males had more NK1.1^+^ lymphocytes under the same conditions, indicating a significant sexual dimorphic pattern that persisted following infection with the parasite (Figure 4A,C). Similarly, a dimorphic pattern was observed in the number of CD8^+^ T lymphocytes; in the absence of infection, females had a greater number of these cells than males did. However, unlike the findings for NK1.1^+^ cells, males maintained similar CD8^+^ cell numbers following infection with *P. berghei* ANKA (Figure 4B,D).

Furthermore, given that the host’s defense against *P. berghei* ANKA depends on the synthesis of IL-2 and IFN-γ, which are cytokines that promote the activation of NK cells [17], we quantified their plasma concentrations. As the testosterone concentration significantly increased in the infected group treated with letrozole, the IFN-γ concentration also increased; this concentration was significantly greater in males than in females under the same conditions, resulting in a sexually dimorphic pattern. However, the increase in free testosterone induced by LT reduced the concentration of IFN-γ in both sexes, eliminating the differences observed between males and females, suggesting a probable testosterone-concentration-dependent effect (Figure 4E). Finally, in infected males, the treatments did not affect the concentrations of testosterone. In contrast, treatment with letrozole or testosterone increased IL-2 concentrations in infected females, once again demonstrating a sexually dimorphic pattern in infected mice treated with testosterone. However, the combination of LT again significantly reduced the IL-2 concentration in infected females, thereby eliminating the dimorphic pattern (Figure 4F).

### 2.5. Testosterone Increased the Number of Macrophages in Infected Females and Modulated the Concentrations of TNF-α, IL-17A and IL-10 in a Sexually Dimorphic Manner

Given that the increase in testosterone concentration was associated with a decrease in the levels of IL-2 and IFN-γ, cytokines involved in the activation of effector cells and in the elimination of *Plasmodium* [18], we investigated whether changes in the concentration of this hormone altered the number of macrophages and the concentrations of IL-17A and TNF-α, given that macrophages phagocytose the parasite [19] and that IL-17 plays a role in the recruitment of phagocytes to eliminate the parasite and contributes to its pathology [9,20]. Uninfected females exhibited a significantly greater number of macrophages than males did in all the groups, generating a dimorphic pattern. Furthermore, the administration of letrozole, testosterone, or the combination of LT increased the number of macrophages in infected females, whereas in infected males it significantly increased the number of macrophages only in the group treated with testosterone (Figure 5B). In contrast, in females, infection increased the concentration of IL-17A, whereas the combination of LT significantly reduced the concentration of IL-17A in infected males, resulting in a sexually dimorphic pattern under these conditions (Figure 5C).

The concentrations of TNF-α and IL-10 did not significantly change in uninfected males when testosterone levels increased. As expected, infection with *P. berghei* ANKA increased TNF-α concentrations in both sexes. However, the LT combination significantly reduced TNF-α levels in infected females (Figure 5D). Furthermore, the increase in testosterone reduced IL-10 levels in females and males infected with *P. berghei* ANKA (Figure 5E).

### 2.6. The Increase in Testosterone Concentration Led to Dimorphic Patterns in the Number of CD19^+^ Cells and in the Levels of IL-6 and IgM in Infected Females

Infection increases the number of B lymphocytes, the levels of the cytokines IL-4 and IL-6, and antibody levels in males [21]. However, whether these changes affect males and females equally and whether testosterone is responsible for these patterns are not known. Consequently, the CD19^+^ cell population, concentrations of the cytokines IL-4 and IL-6, and levels of IgM and IgG antibodies were quantified. In the absence of infection, compared with females, males exhibited a greater number of CD19^+^ cells, indicating a sexually dimorphic pattern with statistical support. However, infection increased this population exclusively in females, reversing the sexually dimorphic pattern. Furthermore, the increase in testosterone concentration reduced the number of CD19^+^ cells in infected females compared with that in the vehicle-treated group. In contrast, infection in males reduced the number of B lymphocytes, and the increase in testosterone concentration did not significantly alter this population (Figure 6B). With respect to the cytokines IL-4 and IL-6, infected females exhibited significantly higher concentrations when were treated with testosterone. In contrast, compared with vehicle treatment, combined LT treatment markedly reduced the levels of both cytokines (Figure 6C,D).

In terms of IgM antibodies, males presented higher levels than females did, indicating a sexually dimorphic pattern. However, the administration of testosterone or LT significantly increased IgM levels exclusively in females, thereby reversing the dimorphic pattern. In contrast, no changes in IgG levels were detected (Figure 6E), and we did not detect significant differences in IgG subclasses (Supplementary Figure S2).

## 3. Discussion

This study aimed to investigate the role of testosterone in the sexual dimorphism of the immune response in experimental malaria. To this end, we administered testosterone and letrozole, an aromatase inhibitor, to prevent its conversion to estrogens, and infected male and female mice with *P. berghei* ANKA. The effects observed in the group treated with the LT combination were compared with those of the groups that received vehicle, letrozole, and testosterone separately. Therefore, L was compared with the vehicle to assess the independent effect of aromatase inhibition, whereas LT was compared with L to evaluate the effect of testosterone under conditions of aromatase inhibition.

We assessed the hormonal profiles, progression of infection, immune response, and expression of *AR* and *ERα* in the spleen and brain. Overall, our results demonstrated that testosterone differentially modulates the immune response in males and females, leading to sexually dimorphic patterns in hormone concentrations, the inflammatory response, immune cell populations, IgM levels, and the expression of *AR* and *ERα*.

First, we confirmed that the combined administration of letrozole and testosterone to infected animals increased the concentration of free testosterone in both sexes, thereby confirming the validity of our experimental model. Furthermore, in uninfected males, treatment with LT reduced the concentration of 17β-estradiol, as expected, because of the inhibition of aromatase activity [22]. In contrast, this effect was not observed in uninfected females. One possible explanation is that the dose of letrozole used was not sufficient to suppress ovarian estradiol production, thereby maintaining its concentration within the physiological range. These results made it possible to assess the effects of testosterone without significantly altering the estradiol concentration. Taken together, these findings suggest that the regulation of estrogen biosynthesis is more robust in females than in males. In line with these results, *ERα* expression supported the validity of our experimental model. As mentioned previously, aromatase inhibition did not alter 17β-estradiol concentrations in females, suggesting more stringent regulation of the biosynthesis of this steroid. In this context, the reduced availability of estrogens could induce a compensatory increase in *ERα* expression, as described in various experimental models [23].

In this work, the administration of LT tended to reduce *ERα* expression in the spleen, an effect observed exclusively in females infected with *P. berghei* ANKA. It has been reported that letrozole reduces *ERα* expression [24], which could contribute, at least partially, to the decrease observed in this group.

Furthermore, the administration of testosterone and letrozole, both individually and in combination, significantly increased free testosterone concentrations and, accordingly, *AR* expression. These findings suggest that the observed effects may be related, at least in part, to increased androgen signaling. This interpretation is consistent with extensive evidence demonstrating that androgens modulate the function of various immune cell populations primarily through their binding to the *AR* [25,26,27]. Taken together, these findings suggest that the interaction of sex hormones with their receptors is regulated in a sexually dimorphic manner and depends on both the tissue and the hormone concentration.

The infection increased DHEA levels in the females. It is likely that the physiological stress induced by infection led to an increase in the production of glucocorticoids such as cortisol and corticosterone [28]. Corticosterone has been shown to inhibit the activity of the enzyme 3β-steroid dehydrogenase, thereby limiting the conversion of DHEA to androstenedione and of pregnenolone to progesterone. This mechanism could explain, at least in part, the high concentration of DHEA and the low concentration of progesterone in infected females [29]. Furthermore, high concentrations of dehydroepiandrosterone sulfate (DHEA-S) are associated with lower parasitemia and higher hemoglobin concentrations in pubertal women infected with *P. falciparum* [30]. This association could help explain, at least in part, the lower parasitemia and higher hemoglobin concentrations observed in infected females in this study than in infected males. Furthermore, it has been suggested that *AR*-mediated signaling may contribute to a reduction in IL-17A [31] and that IL-17A promotes erythropoiesis and reticulocytosis during infection with *P. berghei* ANKA [32]. Therefore, our results raise the possibility that the increase in testosterone induced by letrozole treatment affects the IL-17A response through mechanisms dependent on androgen signaling and that, secondarily, this alteration contributes to the reduction in hemoglobin concentration during infection in males. However, this interpretation should be considered a mechanistic hypothesis, since in the present study, we did not directly evaluate erythropoiesis, reticulocytosis, or *AR* functional activity.

Interestingly, infected females exhibited a significant increase in body weight compared with uninfected females. Although infection with *P. berghei* ANKA causes weight loss, sex-specific effects have been reported, with distinct variations in females and males [3,9,33]. Manipulation of gonadal steroids has been shown to influence these responses, suggesting a regulatory role for sex hormones in body mass during malaria. Thus, the weight gain observed in infected females may reflect a sex-dependent response and differences in steroid signaling, rather than a general effect of the infection. In addition, we observed that the increase in testosterone concentration was associated with sexually dimorphic patterns in body weight and body temperature. Females treated with letrozole, either alone or in combination with testosterone, exhibited greater weight gain and maintained their body temperature, whereas males showed greater susceptibility to infection-associated changes, characterized by weight loss and a decrease in body temperature.

Weight gain associated with letrozole administration has been previously described in murine models [34], and may be related to metabolic alterations, including changes in glucose metabolism and insulin resistance [35]. Similarly, metabolic alterations, such as increased triglyceride levels [36,37], have been described during infection with *P. berghei* ANKA and could contribute to the differences in metabolic response observed between the two sexes. Taken together, these results demonstrate marked sexual dimorphism in the physiological response to infection and suggest that the effects associated with increased testosterone may depend on both its concentration and the host’s sex. In females, the maintenance of body weight and temperature may reflect compensatory mechanisms that help limit the systemic consequences of infection. These mechanisms could involve, among other factors, the modulation of the immune response, particularly an increase in regulatory cytokines such as IL-10 [38]. However, because these mechanisms were not directly evaluated, further studies are needed to determine whether testosterone-mediated modulation of the immune response contributes to the maintenance of physiological conditions and the greater resistance observed in females during infection.

We subsequently assessed whether the increase in testosterone altered, in a sex-dependent manner, the cell populations involved in parasite clearance. To this end, we quantified the populations of NK1.1^+^ cells and CD8^+^ T lymphocytes, two populations that are major sources of IFN-γ during the immune response to malaria [39]. Our findings revealed that infection reduced the population of NK1.1^+^ cells in both sexes, particularly in females. Similar results have been reported in C57BL/6 mice infected with *P. berghei* ANKA, in which, during the advanced stages of infection, the decrease in NK1.1^+^ cells in the spleen is accompanied by an increase in the NK1.1^+^ and CD4^+^CD8^+^ T-cell populations in the brain, suggesting a process of cell migration toward that organ [40]. Furthermore, infection of C57BL/6 mice with *P. berghei* ANKA reduces the NK1.1^+^ and CD8^+^ cell populations, an effect associated with increased expression of Tim-3 [21,41], an immunoregulatory receptor that controls excessive inflammation [42]. However, overstimulation of this receptor in Th1 cells reduces the concentrations of IFN-γ and IL-2 in malaria [41,43,44]. In this study, an increase in testosterone concentration was associated with a decrease in IFN-γ and IL-10 concentrations, an effect that was particularly evident in males. Although we did not assess Tim-3 expression, our results suggest that testosterone may promote immunoregulatory mechanisms that limit the production of proinflammatory cytokines, possibly via pathways related to this receptor; however, this hypothesis needs to be demonstrated experimentally.

In this study, the increase in testosterone concentration in males was associated with a maintenance of the MAC-3^+^ macrophage population and an increase in TNF-α concentration. These findings suggest that testosterone may modulate the activation status of macrophages, rather than their number, during the immune response to *P. berghei* ANKA.

In contrast, the immune response differed across females. Infection with *P. berghei* ANKA reduced the macrophage population in all groups; however, the increase in testosterone concentration led to an increase in the number of these cells. Our findings suggest that testosterone may play specific immunoregulatory roles in females during infection. Compared with the vehicle-treated females and the group treated with testosterone alone, females treated with the LT combination maintained relatively stable concentrations of IFN-γ, IL-2, IL-17A, TNF-α, IL-10, IL-4 and IL-6. These results suggest that the LT combination is associated with a more balanced cytokine response, thereby preventing excessive changes in inflammatory mediators during infection.

One of the most significant findings was the marked sexually dimorphic pattern in the IL-17 concentration. In males, the LT combination induced a pronounced reduction in the levels of this cytokine, whereas in females, this effect was not evident. Previous studies have identified macrophages as important cellular sources of IL-17 during *P. berghei* ANKA infection [20]. In our study, the reduced IL-17A levels observed in males may have contributed to their poor control of parasitemia. This possibility is supported by evidence from *P. yoelii* 17XL infection, in which macrophages have been identified as the primary cells responsible for controlling parasitemia [45]. Thus, decreased IL-17A may impair macrophage-mediated inflammatory responses and consequently contribute to the increased parasite burden observed in male mice. Our results could have important implications for the control of infection, as IL-17-deficient mice infected with *P. berghei* NK65 developed increased levels of parasitemia, a phenomenon associated with a reduction in splenic macrophages [46]. In addition, the adoptive transfer of macrophages consistently increases IL-17 synthesis via mechanisms dependent on CCL2/7 and IL-23, which are involved in the recruitment and maintenance of these cells [20]. Furthermore, our findings are consistent with those of previous reports, which have shown that dihydrotestosterone promotes the expression of genes associated with an alternative macrophage activation phenotype via *AR*-dependent mechanisms in a model of allergic lung inflammation [47]. Interestingly, we observed an increase in *AR* gene expression in the brains of both males and females. Although we did not assess the phenotype or activation of macrophages in these tissues in this study, these findings suggest that the increase in *AR* expression may contribute to the modulation of the immune response of the brain during *Plasmodium* infection in both sexes; this hypothesis needs to be confirmed in future studies. Furthermore, in the spleens of female mice, the increase in testosterone concentration was associated with increased *AR* expression and increased concentrations of IL-4 and IL-6 during infection. These changes may be related to the modulation of the B-cell response in females and could contribute to antibody production. One possible mechanism could involve the activation of androgen receptor-mediated signaling pathways, as it has been reported that androgens can participate in B-cell differentiation via nongenomic mechanisms, including the activation of pathways such as MAPK, thereby promoting their differentiation into antibody-producing cells [48]. However, serum concentrations of IL-4 and IL-6 increase only in testosterone-treated females; both cytokines are associated with enhanced cellular proliferation and activity mediated by IL-4 [49] and increased IL-6-mediated immunoglobulin secretion [50]. These findings are consistent with the increased IgM levels observed in testosterone-treated females in the present study. The lower number of B cells observed in this group may be explained by the increased differentiation of B cells into antibody-secreting plasma cells or by the enhanced secretory activity of the existing antibody-producing cell population [51]. However, because neither the number nor the functional activity of antibody-secreting cells was evaluated, the specific mechanism underlying the increase in IgM levels cannot be determined. Nevertheless, these findings support the hypothesis that the immune response to testosterone and its associated cytokine profile is sexually dimorphic.

In contrast, infected males showed a reduction in B-cell number, as well as lower concentrations of IL-4 and IL-6 and reduced production of both IgM and IgG antibodies. Notably, IgG synthesis occurs at later stages of the immune response to *Plasmodium* infection; therefore, it is possible that the duration of infection in our model was not sufficient to detect changes in this immunoglobulin. However, the lethality of *P. berghei* ANKA infection prevented us from extending the experimental follow-up without compromising the sample size of the groups. Furthermore, infection reduced *AR* expression in the spleen of males, suggesting that the effects of testosterone on the humoral response do not depend on direct mechanisms mediated by this receptor in B cells. This interpretation is consistent with the findings of previous studies indicating that B-cell development in males does not depend on androgen signaling [52]. Taken together, these findings suggest that the regulation of the humoral response by testosterone follows sexually dimorphic mechanisms that differ between males and females.

In summary, the possible mechanisms mediated by the combination of LT are presented schematically in Figure 7. Our results suggest that, in females, testosterone modulates the immune response by regulating populations such as B lymphocytes and macrophages, most likely via mechanisms dependent on the *AR* and testosterone concentration. In contrast, in males, testosterone appears to regulate the immune response via distinct mechanisms, characterized by the maintenance of the macrophage population and an increase in TNF-α concentration. Taken together, these new findings expand our understanding of the immunoregulatory mechanisms of testosterone and highlight that its effects depend on both sex and hormone concentration.

To our knowledge, this is the most comprehensive study to date to examine in detail the contribution of testosterone to sex differences in the immune response during malaria. Although our findings support a potential role for androgen-mediated mechanisms, the experimental design does not establish a direct causal relationship between increased testosterone after LT treatment and the observed immune changes. Further studies targeting *AR* signaling and tissue steroid metabolism are warranted to clarify the underlying mechanisms involved.

Our findings contribute to a better understanding of the sex-specific effects of testosterone on the immune response during malaria and highlight the need to investigate its potential therapeutic implications in malaria and other infectious diseases. Furthermore, our results underscore the importance of considering the immunological consequences of increased testosterone concentrations, an aspect that has received relatively limited attention in the context of testosterone therapies used for masculinization or muscle mass enhancement.

## 4. Materials and Methods

### 4.1. Mice

In this study, male and female mice of the CBA/Ca strain were used; these were donated by Dr. William Jarra (National Institute for Medical Research, London, UK). The experimental groups consisted of five 12-week-old mice. The mice were housed in a specific-pathogen-free animal facility at the Faculty of Higher Studies (FES) Zaragoza, National Autonomous University of Mexico (UNAM), in strict compliance with Mexican Official Standard NOM-062-ZOO-1999 Especificaciones técnicas para la producción, cuidado y uso de los animales de laboratorio [Technical specifications for the production, care, and use of laboratory animals]. Secretaría de Agricultura, Ganadería, Desarrollo Rural, Pesca y Alimentación: Mexico City, Mexico, 2021 for the production, care and use of laboratory animals. All experimental procedures were approved by the Ethics Committee of the FES Zaragoza UNAM, registration number 28/04/SO/3.4.1. The mice had *ad libitum* access to food and water, were housed under a 12-h light/12-h dark photoperiod, and were acclimatized to the animal facility for 21 days before the start of the treatments. To ensure individual identification and correct experimental allocation, each animal was marked on the tail with a permanent marker with a consecutive number from 1 to 5, and each cage was identified with a specific number throughout the study.

### 4.2. Estrus Synchronization in Females

Fifteen days before the start of treatment, the females were exposed to bedding from the males to synchronize the estrous cycle (Whitten effect) [53]. Subsequently, daily vaginal smears were obtained, and the stage of the estrous cycle was determined by microscopic observation (Carl Zeiss Ltd., Welwyn Garden City, UK) using a 10× objective. The phases of the cycle were identified according to the following cytological criteria: pro-estrus, a predominance of nucleated epithelial cells; estrus, a predominance of cornified epithelial cells; meta-estrus, a predominance of cornified epithelial cells with few nucleated epithelial cells and some leukocytes; and di-estrus, a predominance of leukocytes, with a marked decrease in epithelial cells. Monitoring of the estrous cycle was carried out over 10 days, and the females were concluded to be in the diestrus phase. Only females synchronized in this phase were included in the study; those that did not achieve synchronization were excluded from the experimental groups.

### 4.3. Experimental Design

A large cohort of 12-week-old male CBA/Ca mice, which were randomly allocated to 8 groups (*n* = 5), was used. Two groups were treated with the vehicle (almond oil) for 14 days; only one was infected with 1 × 10^3^ erythrocytes parasitized with *Plasmodium berghei* ANKA, with the day of infection considered day zero; these groups were used as control groups. Two other groups were treated with letrozole for 14 days; only one was infected with the parasite, and treatment with letrozole was continued for an additional 7 days until a total of 21 doses had been administered; letrozole was used to inhibit aromatase enzyme activity. Two further groups received testosterone; treatment began 14 days prior to infection, but the dosage was administered every 72 h, totaling five doses prior to infection, as previously reported [1,3,4]; only one group was infected with *Plasmodium berghei* ANKA; these groups were used to assess the effects of an exogenous increase in testosterone; the remaining two groups were treated with the combination of letrozole and testosterone using the same letrozole and testosterone administration regimens described for each treatment; these groups were used to analyze the effects to increase the availability of testosterone through exogenous administration while simultaneously inhibiting its conversion to estrogens by aromatase.

In addition, eight groups of 12-week-old female CBA/Ca mice were used. The females were first synchronized according to the procedure described above, and then randomly assigned to the same experimental groups and subjected to the same treatment and infection regimens as the males.

All the animals, regardless of their infection status, were weighed daily starting on day 0, and their hemoglobin levels and body temperature were measured through day 8 post-infection. Parasitemia was assessed only in the infected groups of both sexes from 3 through 8 days post-infection.

All the animals were euthanized on day 8 post-infection. Blood was collected to obtain serum for the quantification of sex steroids, cytokines, and antibodies. The spleen was removed, its weight was recorded, and a cell suspension was prepared by mechanical disruption. Splenic cells were used to characterize immune populations by flow cytometry and to extract RNA. The brains were also removed to obtain RNA. RNA from the spleen and brain was used to analyze the mRNA expression of *AR* and *ERα* receptors.

### 4.4. Parasite

The *Plasmodium berghei* ANKA (*P. berghei* ANKA) parasite, originally donated by Dr William Jarra (National Institute for Medical Research, London, UK), was cryopreserved in liquid nitrogen until use. Before each experiment, the parasite was reactivated by passaging it through a 4-week-old CBA/Ca mouse. When parasitemia reached approximately 20%, blood was collected to prepare an inoculum containing 1 × 10^3^ of infected erythrocytes in 100 µL of phosphate-buffered saline (PBS) per mouse. The inoculum was administered intravenously, with this time point being designated as day 0 post-infection. Once the blood had been collected to prepare the inoculum, the donor mouse was euthanized by cervical dislocation.

### 4.5. Administration of Letrozole and Testosterone

Letrozole (Pharmaceutical Secondary Standard; Certified Reference Material; Merck-Sigma Aldrich, St. Louis, MO, USA) was ground in a mortar and suspended in almond oil (Zential, Italy). It was administered subcutaneously at a dosage of 7 mg/kg body weight in a volume of 30 µL of almond oil per mouse, as a daily dose for 14 days before infection and continued until day 6 postinfection (PI), with a total of 21 doses administered according to [5,9].

Testosterone (Sostenon 250 Schering-Plow, Kenilworth, NJ, USA) was administered subcutaneously at a dosage of 30 mg/kg body weight, diluted in 30 µL of almond oil, every 72 h for 3 weeks before infection, for a total of 5 doses before infection, in accordance with the protocol described by Benten et al. [54]. Mice treated with the combination of letrozole and testosterone (LT) received both compounds at the same doses via the same route of administration and according to the same treatment regimen as described previously [5,9].

### 4.6. Quantification of Steroid Concentrations

On day 8 PI, blood was collected into tubes containing PBS supplemented with 5% heparin as an anticoagulant. The samples were subsequently centrifuged at 1000× *g* for 5 min to separate the plasma. Subsequently, 100 µL of plasma was taken and diluted with 900 µL of PBS, and the sex steroids were extracted using liquid–liquid extraction with ethyl ether (JT Baker, Fisher Scientific, St. Louis, MO, USA). Following extraction, the aqueous phase was frozen on dry ice to facilitate recovery of the organic phase, which was transferred to a clean tube and evaporated completely in a water bath at 37 °C. Finally, the dry residue was reconstituted in 1000 µL of 0.1% gelatin-PBS for subsequent quantification.

#### 4.6.1. Free Testosterone

The concentration of free testosterone was determined using an enzyme-linked immunosorbent assay (EIA) with a Free Testosterone EIA-2924 kit (DRG International, Marburg, Germany) in accordance with the manufacturer’s instructions. Twenty-five microliters of the extract from each sample and 25 µL of the free testosterone enzyme conjugate were added to the wells of an ELISA plate and incubated for 1 h at 37 °C. The wells were then washed four times, and 100 µL of tetramethylbenzidine substrate solution (H_2_O_2_/TMB 0.26 g/L) was added; the plate was incubated for 25 min at room temperature in the dark. The reaction was stopped by the addition of stop solution, and the plate was gently shaken before the absorbance was measured at 450 nm using a MultiSkan Ascent 96 plate reader (Thermo Fisher Scientific, Waltham, MA, USA). Free testosterone concentrations were calculated from the standard curve provided with the kit.

#### 4.6.2. 17β-Estradiol

The concentrations of the previously obtained extracts were determined using a highly sensitive chemiluminescent immunoassay with an Immulite Estradiol High Sensitivity (LKE2) kit (Siemens Healthineers, Llanberis, Gwynedd, UK) in accordance with the manufacturer’s instructions. For each determination, 500 µL of the sample extract was used. The concentrations of 17β-estradiol were quantified automatically on the Immulite 1000 Immunoassay System (Siemens Healthineers, UK).

#### 4.6.3. Dehydroepiandrosterone (DHEA)

Once the sex steroid extract was prepared, an aliquot was taken to determine the DHEA concentration using a commercial enzyme-linked immunosorbent assay (EIA-3415 kit, DRG International, Marburg, Germany). Ten microliters of sample or standard were added to a 96-well plate, mixed with conjugate (100 µL), and incubated at 37 °C. The plate was then washed, and tetramethylbenzidine (TMB) substrate solution was added, followed by a second incubation at 37 °C. The reaction was stopped with stop solution (H_2_SO_4_), and the absorbance was measured at 450 nm using a MultiSkan Ascent reader (Thermo Fisher Scientific, Waltham, MA, USA). DHEA concentrations were calculated from the standard curve provided in the kit.

#### 4.6.4. Progesterone

The concentration was determined by an enzyme-linked immunosorbent assay (EIA) using a Progesterone EIA-1561 kit (DRG Diagnostic, DRG International, Marburg, Germany; sensitivity: 0.045 ng/mL) in accordance with the manufacturer’s instructions. A 25 µL amount of the standard or the extract from each sample was added to the wells of an ELISA plate and incubated for 5 min at room temperature. Afterward, 200 µL of the enzyme conjugate was added, the plate was shaken, and it was incubated for 1 h at room temperature. Following the washing step, 200 µL of TMB substrate was added to each well, and the plate was incubated in the dark at 25 °C for 15 min. The enzymatic reaction was halted by adding 100 µL of stop solution. The absorbance was read at 450 nm using a MultiSkan Ascent 96 microplate reader (Thermo Fisher Scientific, Waltham, MA, USA). Progesterone levels were determined based on the standard calibration curve supplied with the assay kit.

### 4.7. RNA-to-cDNA Reverse Transcription

On day 8 PI, the spleen and brain were collected from each mouse. Total RNA was isolated from each tissue sample using 1 mL of TRIzol reagent (Invitrogen, Thermo Fisher Scientific, Carlsbad, CA, USA) and 0.2 mL of chloroform: isoamyl alcohol (49:1) (Sigma-Aldrich). The aqueous phase was separated, and the RNA was precipitated with an equal volume of isopropyl alcohol (Sigma-Aldrich). The samples were subsequently washed with 80% ethanol, centrifuged at 13,100× *g* at 4 °C, air-dried, and resuspended in nuclease-free water. The concentration and purity of the RNA were determined by measuring the absorbance at 260 nm using a MultiSkan Go microvolume reader (Thermo Fisher Scientific, Waltham, MA, USA). For the synthesis of complementary DNA (cDNA), 5 µg of total RNA was mixed with 1 µL of oligo(dT)_15_ primer (0.5 µg) (Invitrogen, Thermo Fisher Scientific) and incubated at 65 °C for 10 min in a thermocycler (Mastercycler Gradient, Eppendorf, Hamburg, Germany), and the reaction was stopped on ice. First-strand buffer, DTT (0.1 M), 5 mM dNTPs, RNase inhibitor (40 U/µL), and reverse transcriptase enzyme (MMLV-RT 200 U/µL; Promega, Madison, WI, USA) were subsequently added. The reverse transcription reaction was carried out at 37 °C for 1 h, followed by incubation at 95 °C for 5 min. The concentration of cDNA was adjusted to 15 ng/µL using sterile, nuclease-free water for use in subsequent gene expression analyses.

### 4.8. Quantification of the Relative Expression of Androgen Receptor (AR) and Estrogen Receptor α (ERα)

The expression of *AR* and *ERα* was determined by quantitative PCR (qPCR) using an Applied Biosystems 7500 Real-Time PCR thermocycler (Thermo Fisher, Baltics, Vilnius, Lithuania). The sequences of the primers used were as follows: *AR* FW 5′-TGGGACTGATGGTATTTG-3′, *AR* RV 5′-ATGCGGTACTCATTGAAA-3′, *ERα* FW 5′-GCACACTAAGAAGAATAGC-3′, *ERα* RV 5′-GAAGGATCATATTCAGAATAGA-3′, *ERα* TaqMan probe 5′-(6FAM) CATCCAACAAGGCACTGACCATC (BHQ1)-3′, *GAPDH* FW 5′-TCGGTGTGAACGGATTTGG-3′, *GAPDH* RV 5′-TCTCCACTTTGCCACTGC-3′ and TaqMan probe 5′-(6FAM) CGTATTGGGCGCCTGGTCACCAGGG (BHQ1)-3′. For RNA expression analysis, 30 ng/µL of cDNA from each sample was used, along with forward and reverse primers at 300 nM, SYBR Green/ROX qPCR MasterMix (Thermo Fisher, Baltics, Vilnius, Lithuania), and ROX (50 nM) (Invitrogen, Thermo Fisher Scientific) in a final volume of 5 µL. *AR* amplification was performed for 45 cycles following a 5-min precycle at 95 °C, with denaturation at 95 °C for 30 s, hybridization at 51.4 °C for 45 s, and extension at 72 °C for 45 s. *ERα* expression was determined using a TaqMan system with 15 ng/µL cDNA, PCR buffer, 2 mM dNTPs, magnesium sulfate (3.5 mM), 100 nM primers, a TaqMan probe (100 nM) and GoTaq G2 Flexi enzyme at 0.25 U/µL (Promega). *ERα* amplification was performed for 40 cycles following a 5-min precycle at 94 °C, with denaturation at 94 °C for 30 s, hybridization at 50.0 °C for 45 s, and extension at 72 °C for 45 s. Positive and negative controls were included in each assay and previously evaluated on a 10% polyacrylamide gel, and relative gene expression was calculated using the 2^−ΔΔCt^ method, with *GAPDH* as the reference gene.

### 4.9. Parasitemia

From day 3 PI onward, parasitemia was assessed daily using blood smears prepared from a drop of blood taken from the caudal vein. The smears were fixed with absolute methanol (Sigma-Merck, St. Louis, MO, USA) and stained with Giemsa (Sigma-Merck). The percentage of parasitemia was determined by counting infected erythrocytes using an optical microscope (Carl Zeiss Ltd., Welwyn Garden City, UK) with a 100× immersion objective. The results are expressed as the percentage of parasitized erythrocytes.

### 4.10. Measurement of Changes in Body Weight and Body Temperature

The body weights of the mice were recorded daily from day 0 post-infection (PI) to day 8 PI, always at the same time, using a semianalytical balance (Ohaus, Parsippany, NJ, USA). Body temperature was measured using an infrared thermometer (Thermofocus^®^, 01500A/H1N1, Vedano Olana-Varese, Italy). Changes in body weight and temperature were calculated relative to the values recorded on day 0 PI using Formula (1).

(1) $$Change\;in\;body\;mass\;or\;temperature=\frac{Day\;PI\times 100}{Day\;0\;PI}-100$$

### 4.11. Hemoglobin Quantification

The hemoglobin concentration was determined daily by drawing 2 µL of blood from the caudal vein of each mouse. Each sample was mixed with 498 µL of Drabkin’s reagent (Sigma) and incubated in the dark for 5 min at room temperature. Afterward, 100 µL of the mixture was transferred to a 96-well plate, and the absorbance was recorded at 540 nm using a MultiSkan Go plate reader (Thermo Fisher Scientific). The hemoglobin concentration was calculated by interpolation from a standard curve of known concentrations.

### 4.12. Splenic Index

The splenic index was determined on day 8 PI as a measure of inflammation. Following euthanasia, the spleen was removed and weighed using an analytical balance (Sartorius, Göttingen, Germany), and this value was divided by the mouse weight before euthanasia. This calculation was performed for each experimental subject, and the mean ± SEM for each group was reported.

### 4.13. Quantification of Cell Populations

On day 8 PI, the spleens were aseptically removed and mechanically disrupted on a nylon mesh using 500 µL of sterile PBS. The suspension was subsequently centrifuged at 700× *g* for 5 min at 4 °C. Afterward, 200 µL of the suspension was incubated with lysis and fixation solution (BD Biosciences, Franklin Lakes, NJ, USA) to remove erythrocytes. The cells were counted in a Neubauer chamber and mixed with staining buffer (PBS, bovine serum albumin and 0.1% sodium azide). To identify cell populations by flow cytometry, three panels of fluorophore-conjugated anti-mouse monoclonal antibodies were prepared. Panel 1: FITC-conjugated anti-CD3, APC-conjugated anti-CD4 and PE-conjugated anti-CD8 antibodies were used to identify helper T lymphocytes (CD3^+^CD4^+^) and cytotoxic T lymphocytes (CD3^+^CD8^+^). Panel 2: FITC-conjugated anti-CD3, APC-conjugated anti-CD19 and PE-conjugated anti-Mac-3 antibodies to identify B lymphocytes (CD19^+^) and macrophages (Mac-3^+^). Panel 3: FITC-conjugated anti-CD3 and PE-conjugated anti-NK1.1 antibodies were used to identify NK cells (CD3^−^NK1.1^+^). All antibodies were obtained from BioLegend (San Diego, CA, USA). (1 × 10^6^) cells were incubated with the antibodies for 30 min in the dark and then were washed with FACS solution, and 10,000 events per sample were acquired on a FACSAria II flow cytometer (BD Biosciences, San José, CA, USA). Analysis was performed by initially selecting the leukocyte population on the basis of its forward and side scatter properties (FSC and SSC), followed by the exclusion of cell aggregates using singlet gates. Subsequently, populations of helper T lymphocytes (CD3^+^CD4^+^), cytotoxic T lymphocytes (CD3^+^CD8^+^), B lymphocytes (CD19^+^), macrophages (Mac-3^+^) and NK cells (CD3^−^NK1.1^+^) were identified. The data are expressed as the number of cells per 10,000 events acquired.

### 4.14. Cytokine Quantification

To quantify the cytokine concentrations, the animals were euthanized on day 8 post-infection, whole blood was collected into heparinized tubes and the plasma was separated by centrifugation at 200× *g* for 5 min at 4 °C. For cytokine quantification, 50 μL of plasma was mixed with 50 μL of PE-labeled capture beads and detection reagent from the Mouse Th1/Th2/Th17 Cytometric Bead Array (CBA) kit (BD Biosciences, San Jose, CA, USA). The samples were washed by centrifugation at 200× *g* for 5 min and resuspended in FACS solution (BD Biosciences). The samples were analyzed using a FACSAria II flow cytometer (BD Biosciences). The concentrations of IL-2, IL-4, IL-6, IL-10, IL-17A, IFN-γ, and TNF-α were determined on the basis of the standard curves provided with the kit, and data were analyzed using FCAP Array™ v3.0 software (BD Biosciences).

### 4.15. Quantification of Antibodies

Antibodies specific to *Plasmodium berghei* ANKA were quantified using indirect ELISA. The plates were coated with 100 µL of *P. berghei* ANKA antigen solution (10 µg/mL) prepared in carbonate buffer (pH 9.6) and incubated for 12 h at 4 °C. The plates were subsequently washed four times with 0.05% Tween 20 containing PBS, and non-specific binding sites were blocked with 200 µL of a solution containing 10% normal serum and 3% skim milk in carbonate buffer and incubated for 2 h at 37 °C. The plates were subsequently washed, and 100 µL of mouse serum diluted 1:20 in PBS/1% bovine serum albumin/0.05% Tween was added. The plates were incubated for 2 h at 37 °C. The plates were washed, and goat anti-mouse antibodies conjugated to horseradish peroxidase specific for IgG1, IgG2a, IgG2b, IgG3, total IgG or IgM (Zymed, San Francisco, CA, USA), diluted to previously standardized concentrations, were added. The plates were subsequently washed and incubated with a streptavidin-peroxidase solution (diluted 1:3000 in PBS/0.05% Tween 20). They were subsequently washed again and incubated with ortho-phenylenediamine (OPD, 0.4 mg/mL) in citrate buffer (pH = 5.0) containing 0.03% hydrogen peroxide and incubated in the dark for 20 min. The reaction was stopped with 100 µL of 1 N sulfuric acid, and the absorbance was measured in a microplate reader at 450 nm. The results were normalized against an internal standard of CBA/normal mouse serum, and in the absence of standards for each isotype, the results are expressed as the absorbance.

### 4.16. Statistical Analysis

The sample size (*n*) was determined by considering the ethical principles governing *in vivo* experimentation, with the aim of obtaining results with adequate statistical validity while using the smallest possible number of mice per group, ensuring that (*n* = 5) provides statistically relevant evidence. To summarize changes in hemoglobin concentration, body weight, and body temperature during the experimental period (days 0–8 post- infection), the area under the curve (AUC) was calculated for each group. All variables were analyzed using a two-way ANOVA. To minimize error inflation and avoid false positives associated with the small number of replicates, *p*-values were adjusted using the Benjamini–Hochberg method (*p* ≤ 0.05) [55]. The corrected *p*-values and the degrees of freedom are provided in the Supplementary Table S1. All the statistical analyses were performed using GraphPad Prism version 9.5.0 (GraphPad Software, Boston, MA, USA).

## 5. Conclusions

Overall, our findings suggest that testosterone modulates immune responses in males and females during infection with *P. berghei* ANKA in a concentration- and sex-dependent manner. In males, it promotes mechanisms associated with a reduction in IL-17A levels, the maintenance of macrophages, and an increase in TNF-α levels; however, in females, it may contribute to an increase in macrophage populations and a more efficient B-cell response, mechanisms suggested to be promoted by interactions with its androgen receptor, which would partly explain the sexual dimorphism observed during infection.

Taken together, our findings reveal that testosterone is a key regulator of sexually dimorphic immune responses during *P. berghei* ANKA infection, identifying sex as a major determinant of host immunity. This study provides further insight into the sex-specific effects of testosterone on immune responses and disease severity during malaria, emphasizing the importance of considering sex as a key biological variable in the regulation of immunity. These findings also support further investigation into the potential therapeutic implications of testosterone modulation in malaria and other infectious diseases.

## Figures and Tables

**Figure 1:**
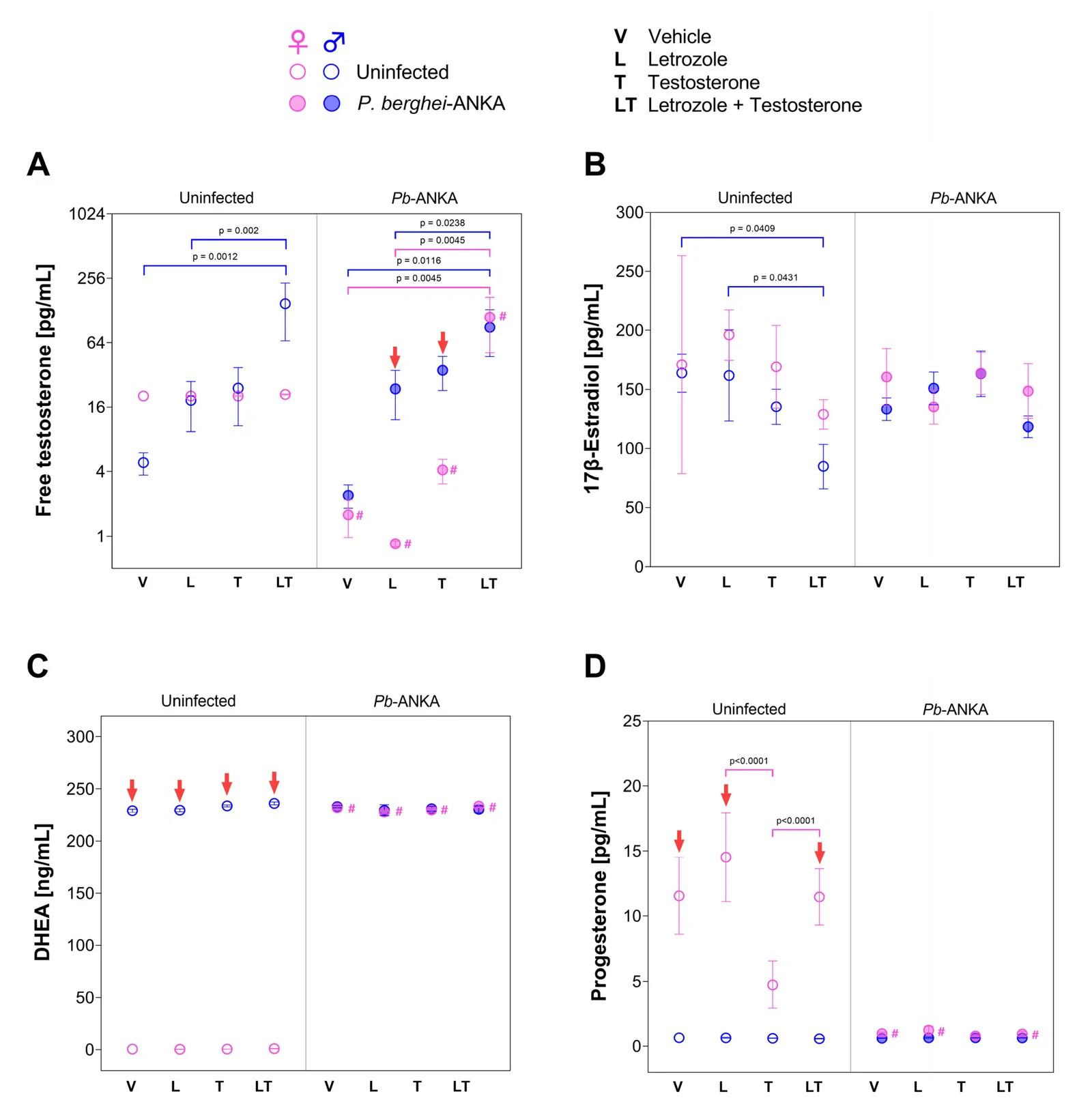
Treatment with LT increased the concentration of free testosterone in male and female mice infected with *P. berghei* ANKA. Four groups of female CBA/Ca mice (pink circles) and four groups of male mice (blue circles) were treated with vehicle (V), letrozole (L), testosterone (T), or a combination of both (LT). In each group, half the animals were infected with *P. berghei* ANKA (filled circles) and the other half remained uninfected (empty circles). On day 8 PI, the mice were euthanized, blood was collected, and the plasma concentrations of (**A**) free testosterone; (**B**) 17β-estradiol; (**C**) dehydroepiandrosterone (DHEA); and (**D**) progesterone were determined. Each circle represents the mean for each group of mice ± SEM (*n* = 5). The graphs show the statistical analysis used to determine: the effect of the treatment on each sex (specific *p*-values), the effect of the infection on each sex (#), and the dimorphic effect between treatments (red arrows). Based on these considerations, a statistical analysis was performed using two-way ANOVA. Significant differences were defined as *p* ≤ 0.05 and are indicated in blue for significant comparisons among males and in pink for comparisons among groups of females. Owing to the number of groups and the number of repetitions, *p*-values were adjusted using the Benjamini–Hochberg test. The specific *p*-values for the comparisons marked with # and red arrows, as well as the F-values and degrees of freedom (DF), are reported in Supplementary Table S1.

**Figure 2:**
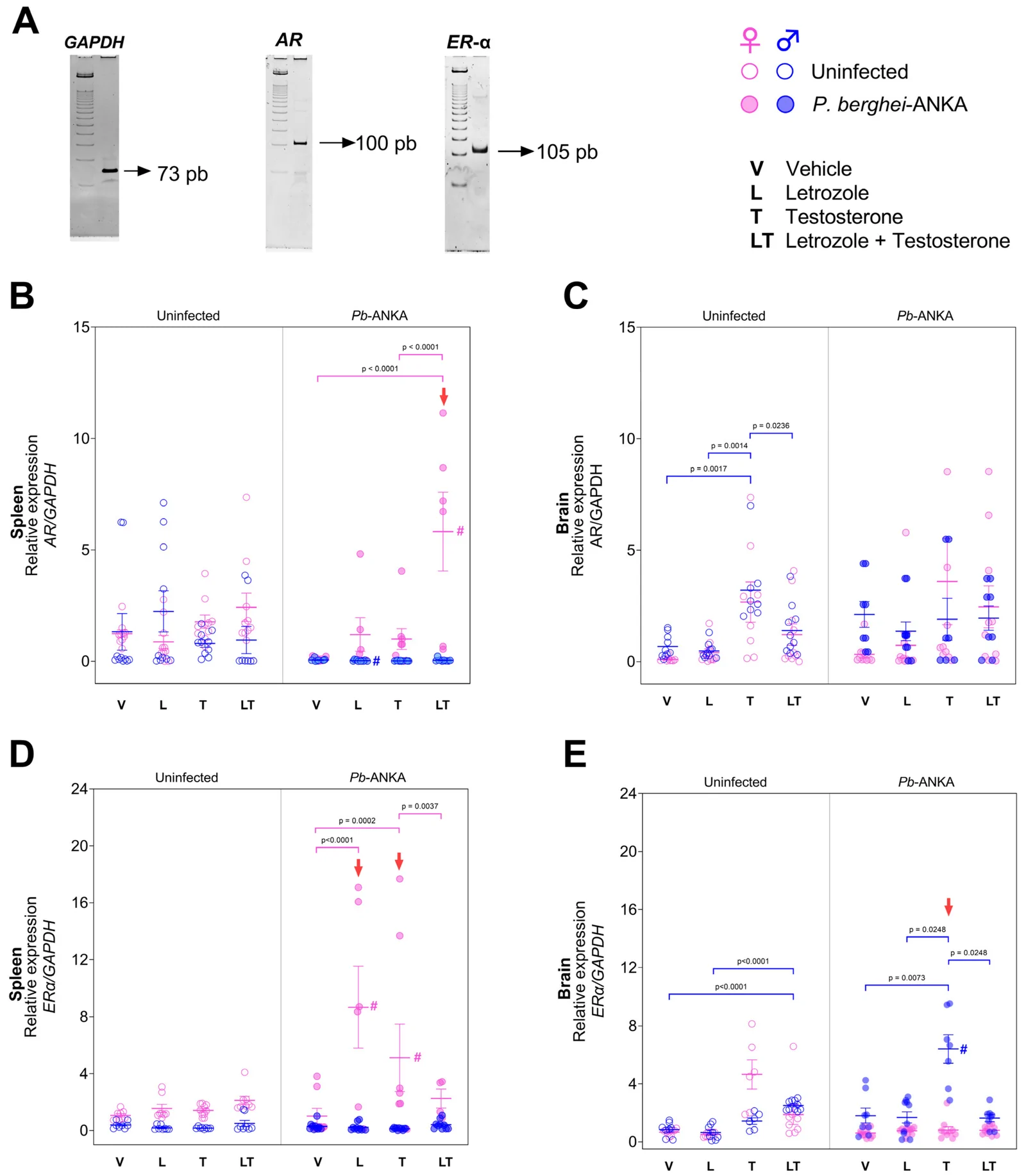
*AR* and *ERα* expression in the spleen was dependent on testosterone concentration in females. In contrast, testosterone administration increased *ERα* expression in the brains of infected males. Four groups of female CBA/Ca mice (pink circles) and four groups of male mice (blue circles) were treated with vehicle (V), letrozole (L), testosterone (T) or a combination of letrozole and testosterone (LT). Half of the mice in each group were infected with *P. berghei* ANKA (filled circles) and the other half were not infected (empty circles). (**A**) Polyacrylamide gel of the positive control for *GAPDH*, *AR*, and *ERα*. On day 8 PI, the mice were euthanized and mRNA was isolated from the spleen and brain; this was reverse-transcribed into cDNA, and the expression of (**B**,**C**) *AR* and (**D**,**E**) *ERα* was measured by real-time PCR. Each circle represents the expression of the receptor of each mouse ± SEM (*n* = 5). The graphs show the statistical analysis used to determine the effect of the treatment on each sex (specific *p*-values), the effect of the infection on each sex (#), and the dimorphic effect between treatments (red arrows). On the basis of these considerations, a statistical analysis was performed using two-way ANOVA. Significant differences were defined as *p* ≤ 0.05 and are indicated in blue for significant comparisons among males and in pink for comparisons among groups of females. Owing to the number of groups and the number of repetitions, *p*-values were adjusted using the Benjamini–Hochberg test. The specific *p*-values for the comparisons marked with # and red arrows, as well as the F-values and degrees of freedom (DF), are reported in Supplementary Table S1.

**Figure 3:**
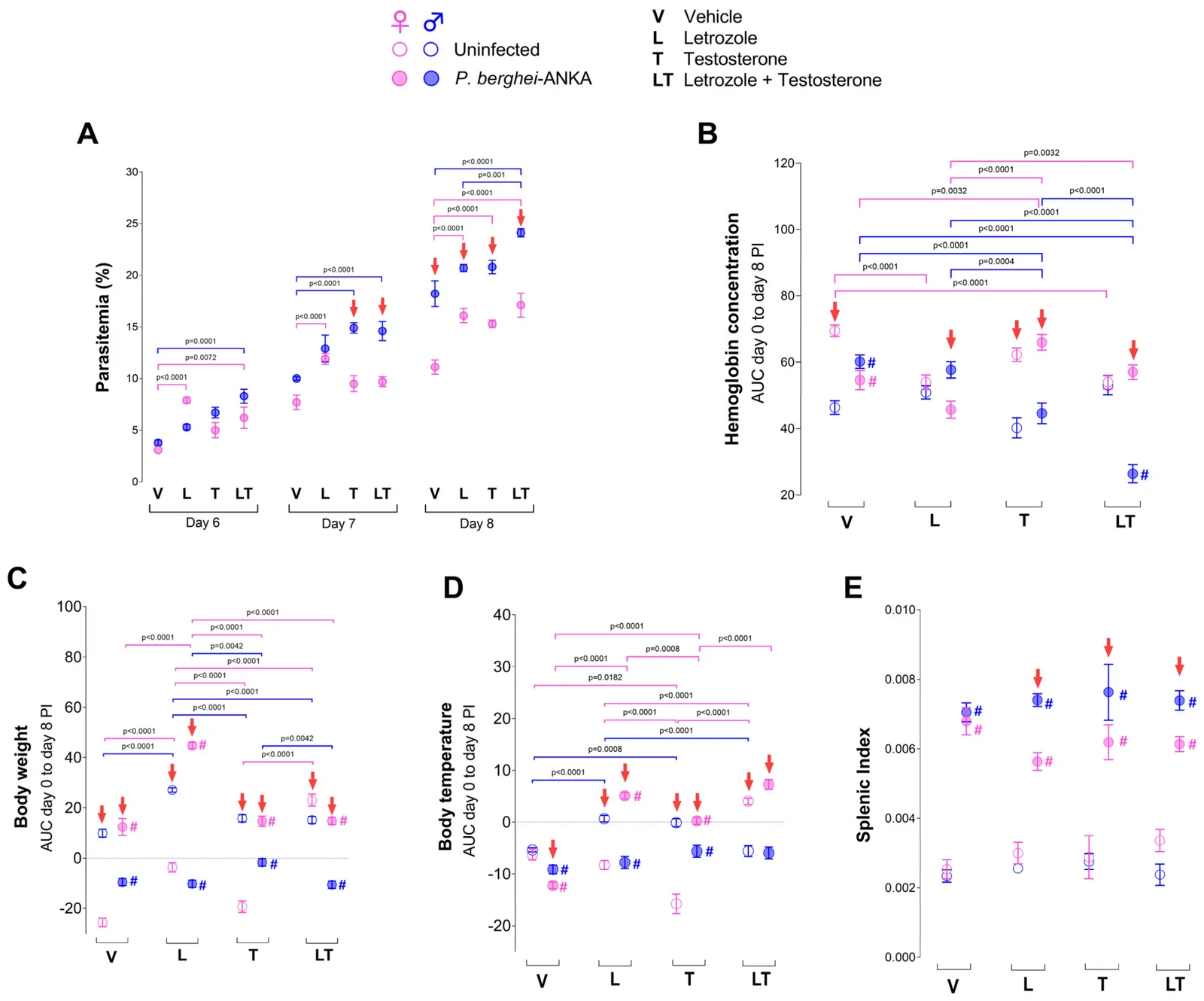
Increasing testosterone concentrations were associated with increased disease severity in males, while modulating body weight and temperature in infected females. Four groups of female CBA/Ca mice (pink circles) and four groups of male mice (blue circles) were treated with vehicle (V), letrozole (L), testosterone (T), or a combination of letrozole and testosterone (LT). In each group, half of the animals were infected with *P. berghei* ANKA (filled circles), whereas the remaining animals were uninfected (open circles). The following parameters were assessed: (**A**) parasitemia on days 6, 7, and 8 PI; the area under the curve (AUC) for (**B**) hemoglobin concentration; (**C**) body weight, and (**D**) body temperature from day 0 to day 8 PI; and (**E**) splenic index in each group. Each data point represents the mean ± SEM (*n* = 5). The graphs show the statistical analysis used to determine: the effect of the treatment on each sex (specific *p*-values), the effect of the infection on each sex (#), and the dimorphic effect between treatments (red arrows). Based on these considerations, a statistical analysis was performed using two-way ANOVA. Significance was defined as *p* ≤ 0.05 and is indicated in blue for significant comparisons among males and in pink for comparisons among groups of females. Owing to the number of groups and the number of repetitions, *p*-values were adjusted using the Benjamini–Hochberg test. The specific *p*-values for the comparisons marked with # and red arrows, as well as the F-values and degrees of freedom (DF), are reported in Supplementary Table S1.

**Figure 4:**
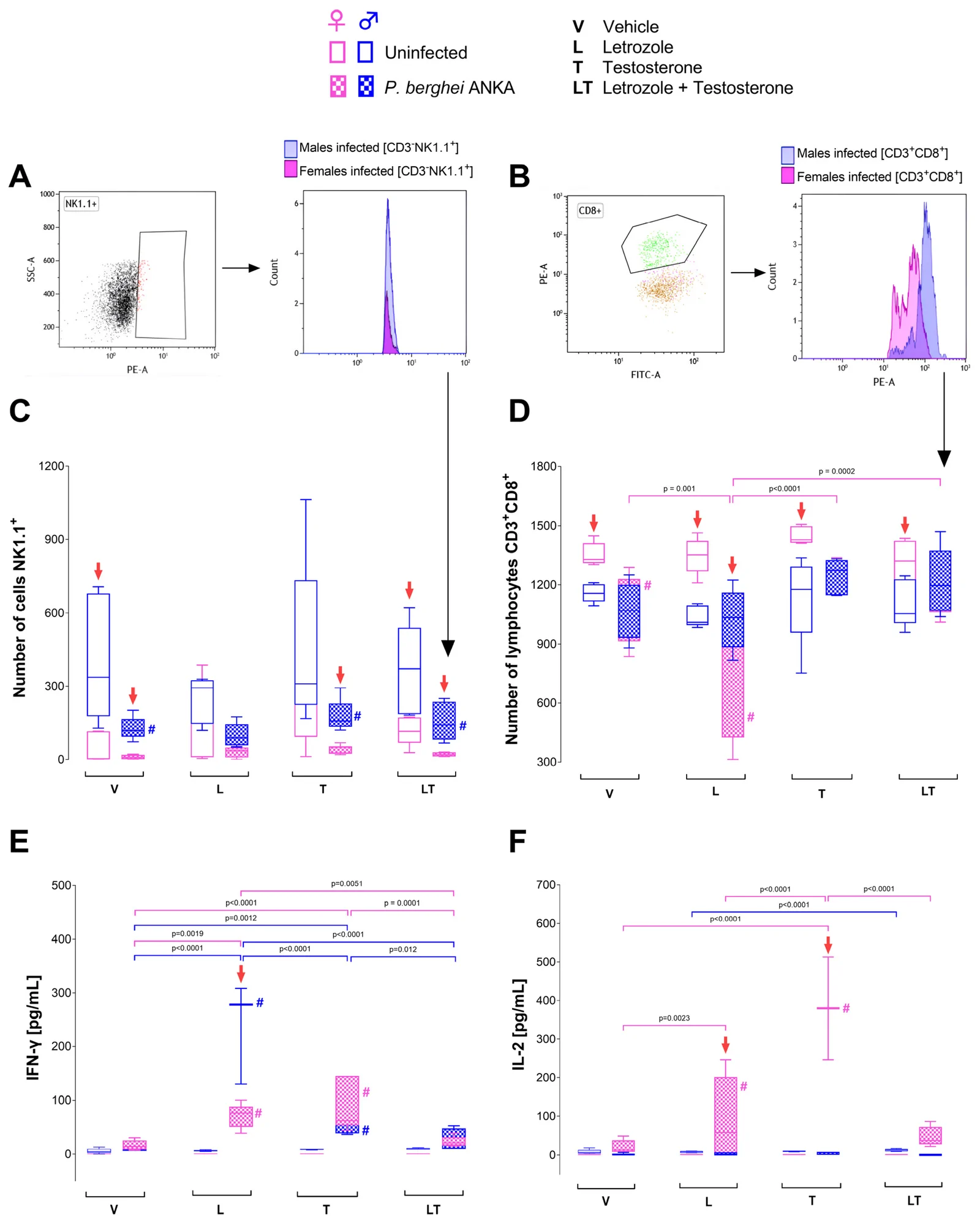
The concentrations of IFN-γ and IL-2 varied according to testosterone levels. Four groups of female CBA/Ca mice (pink bars) and four groups of male mice (blue bars) were treated with vehicle (V), letrozole (L), testosterone (T) or a combination of letrozole and testosterone (LT). Half the animals in each group were infected with *P. berghei* ANKA (hatched bars) and the other half were not infected (unhatched bars). On day 8 PI, all the mice were euthanized and samples of the spleen and blood were collected to quantify the number of NK1.1^+^ and CD3^+^CD8^+^ lymphocytes and the plasma concentrations of IFN-γ and IL-2. (**A**,**B**) Left panel: Gating of the cell population. Right-hand panel: overlay histograms corresponding to the infected group treated with LT. (**C**) Number of NK1.1^+^ lymphocytes. (**D**) Number of CD3^+^CD8^+^ lymphocytes. (**E**) Plasma concentration of IFN-γ and (**F**) plasma concentration of IL-2. Each histogram represents the mean for each group ± SEM (*n* = 5). The graphs show the statistical analysis used to determine: the effect of the treatment on each sex (specific *p*-values), the effect of the infection on each sex (#), and the dimorphic effect between treatments (red arrows); on the basis of these considerations, a statistical analysis was performed using two-way ANOVA. Significant differences were defined as *p* ≤ 0.05 and are indicated in blue for significant comparisons among males and in pink for comparisons among groups of females. Owing to the number of groups and the number of repetitions, *p*-values were adjusted using the Benjamini–Hochberg test. The specific *p*-values for the comparisons marked with # and red arrows, as well as the F-values and degrees of freedom (DF), are reported in Supplementary Table S1.

**Figure 5:**
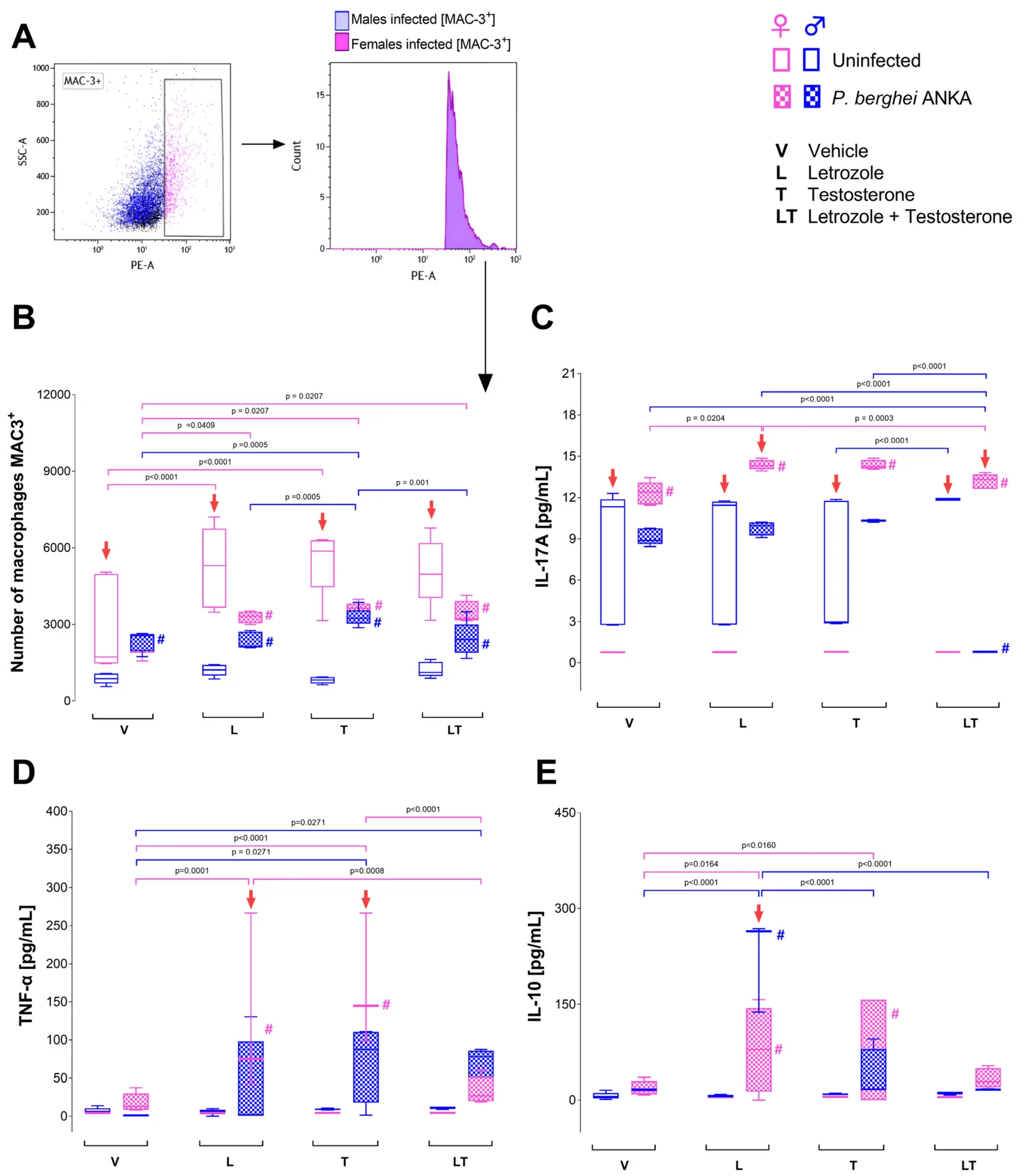
Testosterone increased the number of macrophages in infected females which induced a sex-dependent effect on IL-17A and TNF-α levels. Four groups of female CBA/Ca mice (pink bars) and four groups of male mice (blue bars) were treated with vehicle (V), letrozole (L), testosterone (T) or a combination of both (LT). Half the animals in each group were infected with *P. berghei* ANKA (hatched bars) and the other half were not infected (solid bars). On day 8 PI, all the mice were euthanized, and spleen cells and blood samples were collected to quantify the number of macrophages and cytokine concentrations, respectively. (**A**) Left panel: population selection strategy; right panel: representative overlay histogram of the infected group treated with LT; (**B**) macrophage number (MAC-3^+^); (**C**) plasma concentration of IL-17A; (**D**) plasma concentration of TNF-α; and (**E**) concentration of IL-10. Each bar represents the mean ± SEM (*n* = 5). The graphs show the statistical analysis used to determine: the effect of the treatment on each sex (specific *p*-values), the effect of the infection on each sex (#), and the dimorphic effect between treatments (red arrows); on the basis of these considerations, a statistical analysis was performed using two-way ANOVA. Significant differences were defined as *p* ≤ 0.05 and are indicated in blue for significant comparisons among males and in pink for comparisons among groups of females. Owing to the number of groups and the number of repetitions, *p*-values were adjusted using the Benjamini–Hochberg test. The specific *p*-values for the comparisons marked with # and red arrows, as well as the F-values and degrees of freedom (DF), are reported in Supplementary Table S1.

**Figure 6:**
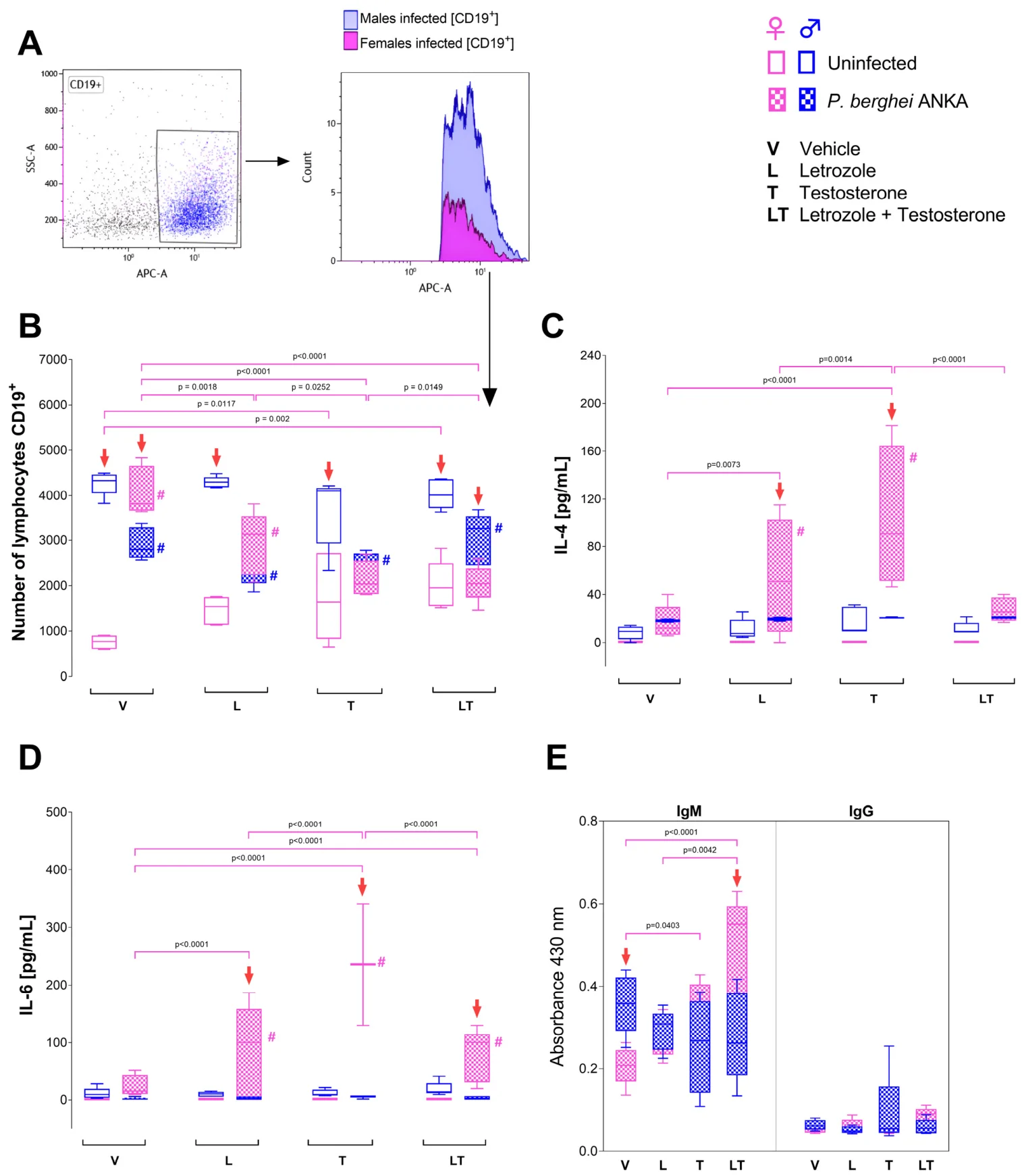
An increase in testosterone concentration reduced the number of CD19^+^ cells in infected females but did not alter that in males, and increased IgM levels in females. Four groups of female CBA/Ca mice (pink bars) and four groups of male mice (blue bars) were treated with vehicle (V), letrozole (L), testosterone (T), or a combination of letrozole and testosterone (LT). Half the animals in each group were infected with *P. berghei* ANKA (hatched boxes), and the other half were not infected (empty bars). On day 8 PI, all the animals were euthanized, and the cells were harvested from the spleen and blood to quantify the numbers of CD19^+^ cells, cytokines, and antibodies. (**A**) Left panel: population selection strategy; right panel: representative overlaid histogram of the infected group treated with LT. (**B**) Number of CD19^+^ cells. (**C**) Plasma concentration of IL-4. (**D**) Plasma concentrations of IL-6 and (**E**) IgM and IgG. Data are expressed as the mean for each group ± SEM (*n* = 5) (**B**–**E**). The graphs show the statistical analysis used to determine: the effect of the treatment on each sex (specific *p*-values), the effect of the infection on each sex (#), and the dimorphic effect between treatments (red arrows); on the basis of these considerations, a statistical analysis was performed using two-way ANOVA. Significant differences were defined as *p* ≤ 0.05 and are indicated in blue for significant comparisons among males and in pink for comparisons among groups of females. Owing to the number of groups and the number of repetitions, *p*-values were adjusted using the Benjamini–Hochberg test. The specific *p*-values for the comparisons marked with # and red arrows, as well as the F-values and degrees of freedom (DF), are reported in Supplementary Table S1.

**Figure 7:**
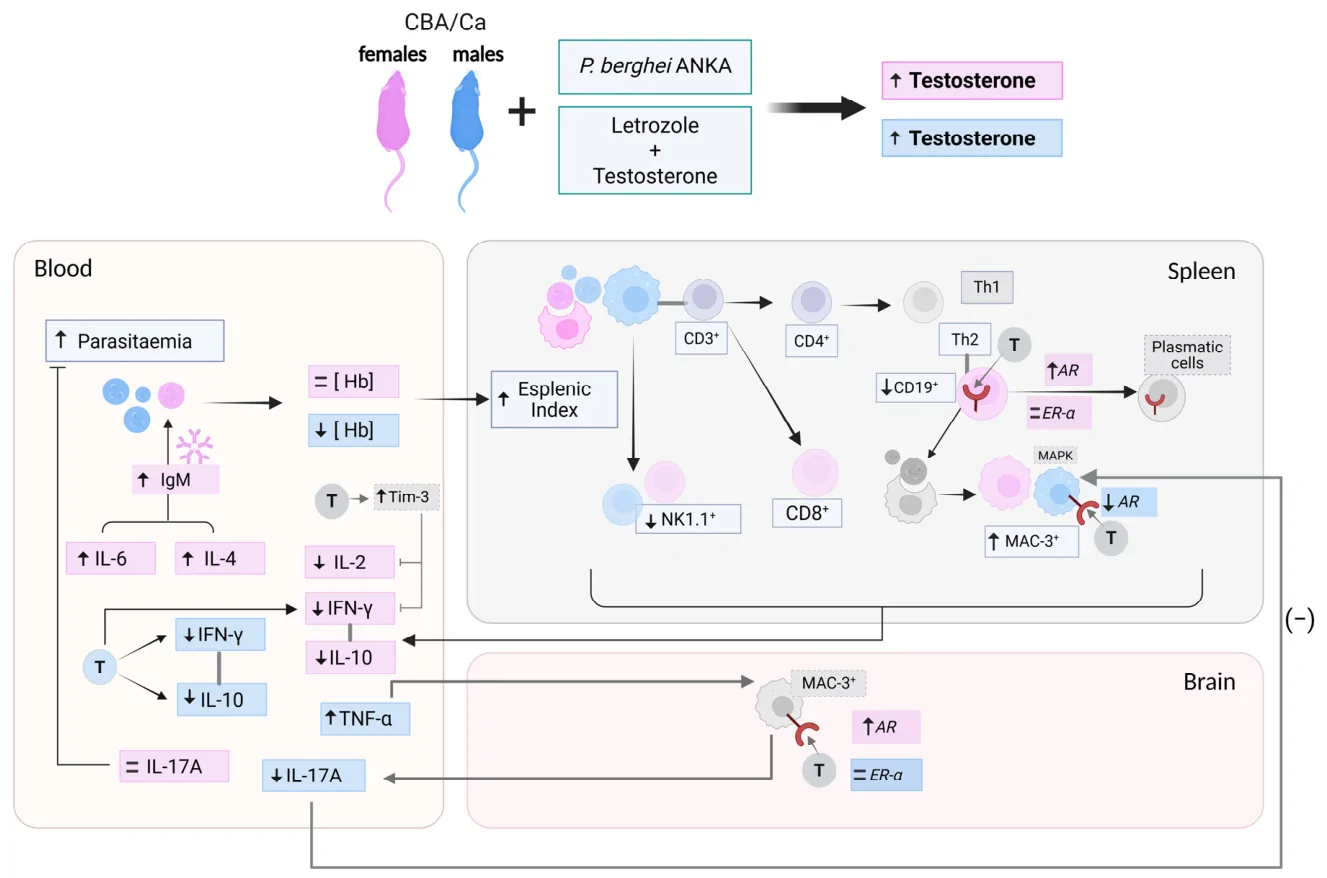
Schematic model of the effects of the combination of letrozole and testosterone (LT) on sexual dimorphism in the immune response during infection with *P. berghei* ANKA. Male and female mice were infected with *P. berghei* ANKA and treated with LT, which increased testosterone levels in both males and females. Although both sexes reached similar levels of free testosterone, compared with females, males exhibited higher parasitemia and lower hemoglobin levels, indicating a sexually dimorphic pattern for both variables. In both sexes, the increase in testosterone concentration reduced the concentrations of IFN-γ and IL-10, suggesting a reduction in inflammation. However, in females only, the increase in testosterone concentrations of IL-6 and IL-4, which are involved in the proliferation and differentiation of B lymphocytes, explains the increase in IgM in this sex, and these antibodies likely facilitated the elimination of the parasite. In contrast, the increase in testosterone concentration increased the number of MAC-3^+^ cells in females, probably in response to the increase in parasitemia. However, the decrease in IL-17A in males was associated with the maintenance of macrophages. Furthermore, increasing testosterone concentration increased androgen receptor expression in the spleen of females, whereas *AR* expression was absent in males, which is likely related to the effect of testosterone on the cell populations of each tissue and to the different immune responses observed in each sex. In the figure, the blue sections correspond to the effects in males; the pink sections represent the effects of increased testosterone in males or females; and the gray sections show the mechanisms suggested to be involved in the regulation of the immune response. The arrows pointing upwards indicate an increase while those pointing downwards indicate a decrease.

## Data Availability

The original contributions presented in this study are included in the article/Supplementary Materials. Further inquiries can be directed to the corresponding author.

## Supplementary Materials

The following supporting information can be downloaded at: https://www.mdpi.com/article/10.3390/ijms27198649/s1.

## Abbreviations

The following abbreviations are used in this manuscript:

*AR*	Androgen Receptor
*ERα*	Estrogen Receptor alpha
DHEA	Dehidroepiandrosterone
IL	Interleukin
IFN	Interferon
LT	Letrozole and Testosterone Treatment
PI	Post-Infection
*P. berghei ANKA*	*Plasmodium berghei* ANKA
TNF	Tumor Necrosis Factor

## References

[1] World Health Organization (2025). World Malaria Report 2025. Addressing the Threat of Antimalarial Drug Resistance.

[2] Landgraf B., Kollaritsch H., Wiedermann G., Wernsdorfer W.H. (1994). Parasite density of Plasmodium falciparum malaria in Ghanaian schoolchildren: Evidence for influence of sex hormones?. Trans. R. Soc. Trop. Med. Hyg..

[3] Aguilar-Castro J., Cervantes-Candelas L.A., Buendía-González F.O., Fernández-Rivera O., Nolasco-Pérez T.d.J., López-Padilla M.S., Chavira-Ramírez D.R., Cervantes-Sandoval A., Legorreta-Herrera M. (2022). Testosterone induces sexual dimorphism during infection with *Plasmodium berghei* ANKA. Front. Cell. Infect. Microbiol..

[4] Benten W., Wunderlich F., Herrmann R., Kühn-Velten W. (1993). Testosterone-induced compared with oestradiol-induced immunosuppression against *Plasmodium chabaudi* malaria. J. Endocrinol..

[5] Nolasco-Pérez T.d.J., Salazar-Castañón V.H., Cervantes-Candelas L.A., Buendía-González F.O., Aguilar-Castro J., Legorreta-Herrera M. (2025). Testosterone modulates oxidative stress in a sexually dimorphic manner in CBA/Ca mice infected with *Plasmodium berghei* ANKA. Int. J. Mol. Sci..

[6] Lu S.-F., McKenna S.E., Cologer-Clifford A., Nau E.A., Simon N.G. (1998). Androgen receptor in mouse brain: Sex differences and similarities in autoregulation. Endocrinology.

[7] Cernetich A., Garver L.S., Jedlicka A.E., Klein P.W., Kumar N., Scott A.L., Klein S.L. (2006). Involvement of gonadal steroids and gamma interferon in sex differences in response to blood-stage malaria infection. Infect. Immun..

[8] Wunderlich F., Marinovski P., Peter W., Benten M., Schmitt-Wrede H.-P., Mossmann H. (1991). Testosterone and other gonadal factor (s) restrict the efficacy of genes controlling resistance to *Plasmodium chabaudi* malaria. Parasite Immunol..

[9] Nolasco-Pérez T.d.J., Cervantes-Candelas L.A., Buendía-González F.O., Aguilar-Castro J., Fernandez-Rivera O., Salazar-Castañón V.H., Legorreta-Herrera M. (2023). Immunomodulatory effects of testosterone and letrozole during *Plasmodium berghei* ANKA infection. Front. Cell. Infect. Microbiol..

[10] Verma R., Krishna A. (2017). Effect of Letrozole, a selective aromatase inhibitor, on testicular activities in adult mice: Both in vivo and in vitro study. Gen. Comp. Endocrinol..

[11] Lew V.L., Tiffert T., Ginsburg H. (2003). Excess hemoglobin digestion and the osmotic stability of *Plasmodium falciparum*–infected red blood cells. Blood.

[12] Krücken J., Mehnert L.I., Dkhil M.A., El-Khadragy M., Benten W.P.M., Mossmann H., Wunderlich F. (2005). Massive destruction of malaria-parasitized red blood cells despite spleen closure. Infect. Immun..

[13] Stevenson M.M., Riley E.M. (2004). Innate immunity to malaria. Nat. Rev. Immunol..

[14] Li C., Corraliza I., Langhorne J. (1999). A defect in interleukin-10 leads to enhanced malarial disease in *Plasmodium chabaudi chabaudi* infection in mice. Infect. Immun..

[15] Ibraheem Y., Bayarsaikhan G., Inoue S.-I. (2023). Host immunity to Plasmodium infection: Contribution of *Plasmodium berghei* to our understanding of T cell-related immune response to blood-stage malaria. Parasitol. Int..

[16] Waki S., Uehara S., Kanbe K., Ono K., Suzuki M., Nariuchi H. (1992). The role of T cells in pathogenesis and protective immunity to murine malaria. Immunology.

[17] Stegmann K.A., De Souza J.B., Riley E.M. (2015). IL-18-induced expression of high-affinity IL-2R on murine NK cells is essential for NK-cell IFN-γ production during murine *Plasmodium yoelii* infection. Eur. J. Immunol..

[18] Setoguchi R., Hori S., Takahashi T., Sakaguchi S. (2005). Homeostatic maintenance of natural *Foxp3*^+^ CD25^+^ CD4^+^ regulatory T cells by interleukin (IL)-2 and induction of autoimmune disease by IL-2 neutralization. J. Exp. Med..

[19] Helegbe G.K., Huy N.T., Yanagi T., Shuaibu M.N., Kikuchi M., Cherif M.S., Hirayama K. (2018). Elevated IL-17 levels in semi-immune anaemic mice infected with *Plasmodium berghei* ANKA. Malar. J..

[20] Ishida H., Imai T., Suzue K., Hirai M., Taniguchi T., Yoshimura A., Iwakura Y., Okada H., Suzuki T., Shimokawa C. (2013). IL-23 protection against *Plasmodium berghei* infection in mice is partially dependent on IL-17 from macrophages. Eur. J. Immunol..

[21] Zhang Y.-W., Su Z.-W., Li Q.-L., Chen R., Jiang N. (2022). The changes of T cells, NK cells and cytokines in mice infected with *Plasmodium berghei* ANKA strain. Acta Vet. Zootech. Sin..

[22] Rashid D., Panda B., Vohora D. (2015). Reduced estradiol synthesis by letrozole, an aromatase inhibitor, is protective against development of pentylenetetrazole-induced kindling in mice. Neurochem. Int..

[23] Marbouti L., Zahmatkesh M., Riahi E., Sadr S.S. (2020). Inhibition of brain 17β-estradiol synthesis by letrozole induces cognitive decline in male and female rats. Neurobiol. Learn. Mem..

[24] Luthra R., Kirma N., Jones J., Tekmal R.R. (2003). Use of letrozole as a chemopreventive agent in aromatase overexpressing transgenic mice. J. Steroid Biochem. Mol. Biol..

[25] Lai J.-J., Lai K.-P., Zeng W., Chuang K.-H., Altuwaijri S., Chang C. (2012). Androgen receptor influences on body defense system via modulation of innate and adaptive immune systems: Lessons from conditional AR knockout mice. Am. J. Pathol..

[26] Ben-Batalla I., Vargas-Delgado M.E., Von Amsberg G., Janning M., Loges S. (2020). Influence of androgens on immunity to self and foreign: Effects on immunity and cancer. Front. Immunol..

[27] Ainslie R.J., Simitsidellis I., Kirkwood P.M., Gibson D.A. (2024). RISING STARS: Androgens and immune cell function. J. Endocrinol..

[28] Gong S., Miao Y.-L., Jiao G.-Z., Sun M.-J., Li H., Lin J., Luo M.-J., Tan J.-H. (2015). Dynamics and correlation of serum cortisol and corticosterone under different physiological or stressful conditions in mice. PLoS ONE.

[29] Topor L.S., Asai M., Dunn J., Majzoub J.A. (2011). Cortisol stimulates secretion of dehydroepiandrosterone in human adrenocortical cells through inhibition of 3βHSD2. J. Clin. Endocrinol. Metab..

[30] Leenstra T., ter Kuile F.O., Kariuki S.K., Nixon C.P., Oloo A.J., Kager P.A., Kurtis J.D. (2003). Dehydroepiandrosterone sulfate levels associated with decreased malaria parasite density and increased hemoglobin concentration in pubertal girls from western Kenya. J. Infect. Dis..

[31] Fuseini H., Yung J.A., Cephus J.Y., Zhang J., Goleniewska K., Polosukhin V.V., Peebles R.S., Newcomb D.C. (2018). Testosterone decreases house dust mite–induced type 2 and IL-17A–mediated airway inflammation. J. Immunol..

[32] Chora Â.F., Marques S., Gonçalves J.L., Lima P., da Costa D.G., Fernandez-Ruiz D., Marreiros M.I., Ruivo P., Carvalho T., Ribeiro R.M. (2023). Interplay between liver and blood stages of Plasmodium infection dictates malaria severity via γδ T cells and IL-17-promoted stress erythropoiesis. Immunity.

[33] Dkhil M.A., Al-Shaebi E.M., Lubbad M.Y., Al-Quraishy S. (2016). Impact of sex differences in brain response to infection with *Plasmodium berghei*. Parasitol. Res..

[34] Arroyo P., Ho B.S., Sau L., Kelley S.T., Thackray V.G. (2019). Letrozole treatment of pubertal female mice results in activational effects on reproduction, metabolism and the gut microbiome. PLoS ONE.

[35] Skarra D.V., Hernández-Carretero A., Rivera A.J., Anvar A.R., Thackray V.G. (2017). Hyperandrogenemia induced by letrozole treatment of pubertal female mice results in hyperinsulinemia prior to weight gain and insulin resistance. Endocrinology.

[36] Chitraju C., Fischer A.W., Ambaw Y.A., Wang K., Yuan B., Hui S., Walther T.C., Farese R.V. (2023). Mice lacking triglyceride synthesis enzymes in adipose tissue are resistant to diet-induced obesity. eLife.

[37] Abdulkareem A.O., Abe E.O., Omilana O.O., Olatunji L.A. (2022). Letrozole suppresses hepatic oxidative stress and ameliorates lipid accumulation in fructose-exposed wister rats. Bull. Pharm. Sci. Assiut Univ..

[38] Li C., Sanni L.A., Omer F., Riley E., Langhorne J. (2003). Pathology of *Plasmodium chabaudi chabaudi* infection and mortality in interleukin-10-deficient mice are ameliorated by anti-tumor necrosis factor alpha and exacerbated by anti-transforming growth factor β antibodies. Infect. Immun..

[39] King T., Lamb T. (2015). Interferon-γ: The Jekyll and Hyde of malaria. PLoS Pathog..

[40] Hansen D.S., Bernard N.J., Nie C.Q., Schofield L. (2007). NK cells stimulate recruitment of CXCR3^+^ T cells to the brain during *Plasmodium berghei*-mediated cerebral malaria. J. Immunol..

[41] Huang B., Liu M., Huang S., Wu B., Guo H., Su X.-Z., Lu F. (2013). Expression of Tim-1 and Tim-3 in *Plasmodium berghei* ANKA infection. Parasitol. Res..

[42] Anderson A.C., Anderson D.E. (2006). TIM-3 in autoimmunity. Curr. Opin. Immunol..

[43] Sánchez-Fueyo A., Tian J., Picarella D., Domenig C., Zheng X.X., Sabatos C.A., Manlongat N., Bender O., Kamradt T., Kuchroo V.K. (2003). Tim-3 inhibits T helper type 1–mediated auto-and alloimmune responses and promotes immunological tolerance. Nat. Immunol..

[44] Liu J., Xiao S., Huang S., Pei F., Lu F. (2016). Upregulated Tim-3/galectin-9 expressions in acute lung injury in a murine malarial model. Parasitol. Res..

[45] Couper K.N., Blount D.G., Hafalla J.C., Van Rooijen N., De Souza J.B., Riley E.M. (2007). Macrophage-mediated but gamma interferon-independent innate immune responses control the primary wave of *Plasmodium yoelii* parasitemia. Infect. Immun..

[46] Barin J.G., Baldeviano G.C., Talor M.V., Wu L., Ong S., Quader F., Chen P., Zheng D., Caturegli P., Rose N.R. (2012). Macrophages participate in IL-17-mediated inflammation. Eur. J. Immunol..

[47] Becerra-Diaz M., Song M., Heller N. (2020). Androgen and androgen receptors as regulators of monocyte and macrophage biology in the healthy and diseased lung. Front. Immunol..

[48] Heinlein C.A., Chang C. (2002). The roles of androgen receptors and androgen-binding proteins in nongenomic androgen actions. Mol. Endocrinol..

[49] Rigley K.P., Thurstan S.M., Callard R.E. (1991). Independent regulation of interleukin 4 (IL-4)-induced expression of human B cell surface CD23 and IgM: Functional evidence for two IL-4 receptors. Int. Immunol..

[50] Nguyen D.C., Garimalla S., Xiao H., Kyu S., Albizua I., Galipeau J., Chiang K.-Y., Waller E.K., Wu R., Gibson G. (2018). Factors of the bone marrow microniche that support human plasma cell survival and immunoglobulin secretion. Nat. Commun..

[51] Shaffer A., Lin K.-I., Kuo T.C., Yu X., Hurt E.M., Rosenwald A., Giltnane J.M., Yang L., Zhao H., Calame K. (2002). Blimp-1 orchestrates plasma cell differentiation by extinguishing the mature B cell gene expression program. Immunity.

[52] Viselli S., Reese K., Fan J., Kovacs W., Olsen N. (1997). Androgens alter B cell development in normal male mice. Cell. Immunol..

[53] Cora M.C., Kooistra L., Travlos G. (2015). Vaginal cytology of the laboratory rat and mouse: Review and criteria for the staging of the estrous cycle using stained vaginal smears. Toxicol. Pathol..

[54] Benten W., Bettenhaeuser U., Wunderlich F., Van Vliet E., Mossmann H. (1991). Testosterone-induced abrogation of self-healing of *Plasmodium chabaudi* malaria in B10 mice: Mediation by spleen cells. Infect. Immun..

[55] Genovese C., Wasserman L. (2002). Operating characteristics and extensions of the false discovery rate procedure. J. R. Stat. Soc. Ser. B Stat. Methodol..

