# Supplementary material for: Testosterone Regulates Sex-Dependent Immune Responses and Androgen Receptor Expression During Plasmodium berghei ANKA Infection
Source: Int J Mol Sci. 2026 Sep 27;27(19):8649. doi: 10.3390/ijms27198649 (PMC13664739; doi:10.3390/ijms27198649)
Supplement: Supplementary file 1 [file ijms-27-08649-s001.zip › Supplementary Table S1.pdf]

Supplementary Table S1. Adjusted p- values for significant comparisons demonstrating the effect of infection in each sex (#) and the dimorphic effect across treatments (red arrows)

Figure	Group	Comparated group	p-adj	F-value (DFn, DFd)
1A. Free testosterone	V females uninfected	V females infected	0.0013	F (6, 43) = 2.007
	L females uninfected	L females infected	<0.0001	F (6, 43) = 2.007
	T females uninfected	T females infected	0.0053	F (6, 43) = 2.007
	LT females uninfected	LT females infected	0.0118	F (6, 43) = 2.007
	L males infected	L females infected	<0.0001	F (1.558, 7.789) = 2.171
	T males infected	T females infected	<0.0001	F (1.558, 7.789) = 2.171
1C. DHEA	V females uninfected	V females infected	<0.0001	F (1.234, 23.45) = 8071
	L females uninfected	L females infected	<0.0001	F (1.234, 23.45) = 8071
	T females uninfected	T females infected	<0.0001	F (1.234, 23.45) = 8071
	LT females uninfected	LT females infected	0.0003	F (1.234, 23.45) = 8071
	V females uninfected	V males uninfected	<0.0001	F (2.186, 13.84) = 0.8484
	L females uninfected	L males uninfected	0.0002	F (2.186, 13.84) = 0.8484
	T females uninfected	T males uninfected	<0.0001	F (2.186, 13.84) = 0.8484
	LT females uninfected	LT males uninfected	<0.0001	F (2.186, 13.84) = 0.8484
1D. Progesterone	V females uninfected	V females infected	<0.0001	F (3, 15) = 29.69
	L females uninfected	L females infected	<0.0001	F (3, 15) = 29.69
	LT females uninfected	LT females infected	<0.0001	F (3, 15) = 29.69
	V females uninfected	V males uninfected	<0.0001	F (3, 40) = 9.755
	L females uninfected	L males uninfected	<0.0001	F (3, 40) = 9.755
	LT females uninfected	LT males uninfected	<0.0001	F (3, 40) = 9.755
2B. *AR* spleen	LT females uninfected	LT females infected	0.0002	F (3, 36) = 8.385
	LT males uninfected	LT females infected	0.0171	F (3, 92) = 7.171
	LT females infected	LT males infected	<0.0001	F (3, 92) = 7.171
2D. *ERα* spleen	L females uninfected	L females infected	<0.0001	F (3, 34) = 9.937
	T females uninfected	T females infected	0.0018	F (3, 34) = 9.937
	L females infected	L males infected	<0.0001	F (3, 82) = 5.474
	T females infected	T males infected	<0.0001	F (3, 82) = 5.474
2D. *ERα* brain	T males uninfected	T males infected	<0.0001	F (3, 119) = 24.16
	T females infected	T males infected	<0.0001	F (3, 119) = 31.44
3A. Parasitemia	T females day 7	T males day 7	<0.0001	F (2, 12) = 441.7
	LT females day 7	LT males day 7	<0.0001	F (2, 12) = 441.7
	V females day 8	V males day 8	<0.0001	F (2, 12) = 441.7
	L females day 8	L males day 8	<0.0001	F (2, 12) = 441.7
	T females day 8	T males day 8	<0.0001	F (2, 12) = 441.7
	LT females day 8	LT males day 8	<0.0001	F (2, 12) = 441.7
3B. Hemoglobin	V females uninfected	V females infected	0.0002	F (3, 64) = 26.70
	V males uninfected	V males infected	0.0003	F (3, 64) = 26.70
	LT males uninfected	LT males infected	<0.0001	F (3, 64) = 26.70
	V males uninfected	V females uninfected	<0.0001	F (3, 64) = 11.55
	L males infected	L females infected	0.0052	F (3, 64) = 11.55
	T males uninfected	T females uninfected	<0.0001	F (3, 64) = 11.55
	T males infected	T females infected	<0.0001	F (3, 64) = 11.55
	LT males infected	LT females infected	<0.0001	F (3, 64) = 11.55
3C. Body weight	V females uninfected	V females infected	<0.0001	F (3, 64) = 77.75
	L females uninfected	L females infected	<0.0001	F (3, 64) = 77.75
	T females uninfected	T females infected	<0.0001	F (3, 64) = 77.75
	LT females uninfected	LT females infected	0.0028	F (3, 64) = 77.75
	V males uninfected	V males infected	<0.0001	F (9, 64) = 53.22

	L males uninfected	L males infected	<0.0001	F (9, 64) = 53.22
	T males uninfected	T males infected	<0.0001	F (9, 64) = 53.22
	LT males uninfected	LT males infected	<0.0001	F (9, 64) = 53.22
	V males uninfected	V females uninfected	<0.0001	F (9, 64) = 53.22
	V males infected	V females infected	<0.0001	F (9, 64) = 53.22
	L males uninfected	L females uninfected	<0.0001	F (9, 64) = 53.22
	L males infected	L females infected	<0.0001	F (9, 64) = 53.22
	T males uninfected	T females uninfected	<0.0001	F (9, 64) = 53.22
	T males infected	T females infected	<0.0001	F (9, 64) = 53.22
	LT males uninfected	LT females uninfected	0.0035	F (9, 64) = 53.22
	LT males infected	LT females infected	<0.0001	F (9, 64) = 53.22
3D. Body temperature	V females uninfected	V females infected	0.0001	F (3, 64) = 50.24
	L females uninfected	L females infected	<0.0001	F (3, 64) = 50.24
	T females uninfected	T females infected	<0.0001	F (3, 64) = 50.24
	V males uninfected	V males infected	0.0157	F (3, 64) = 50.24
	L males uninfected	L males infected	<0.0001	F (3, 64) = 50.24
	T males uninfected	T males infected	0.0002	F (3, 64) = 50.24
	V males infected	V females infected	0.0394	F (9, 64) = 38.37
	L males uninfected	L females uninfected	<0.0001	F (9, 64) = 38.37
	L males infected	L females infected	<0.0001	F (9, 64) = 38.37
	T males uninfected	T females uninfected	<0.0001	F (9, 64) = 38.37
	T males infected	T females infected	<0.0001	F (9, 64) = 38.37
	LT males uninfected	LT females uninfected	<0.0001	F (9, 64) = 38.37
	LT males infected	LT females infected	<0.0001	F (9, 64) = 38.37
3E. Splenic index	V females uninfected	V females infected	<0.0001	F (3, 12) = 0.2122
	L females uninfected	L females infected	<0.0001	F (3, 12) = 0.2122
	T females uninfected	T females infected	<0.0001	F (3, 12) = 0.2122
	LT females uninfected	LT females infected	<0.0001	F (3, 12) = 0.2122
	V males uninfected	V males infected	<0.0001	F (9, 36) = 0.9738
	L males uninfected	L males infected	<0.0001	F (9, 36) = 0.9738
	T males uninfected	T males infected	<0.0001	F (9, 36) = 0.9738
	LT males uninfected	LT males infected	<0.0001	F (9, 36) = 0.9738
	L males infected	L females infected	0.0019	F (9, 36) = 0.9738
	T males infected	T females infected	0.0101	F (9, 36) = 0.9738
	LT males infected	LT females infected	0.0244	F (9, 36) = 0.9738
4C. $NK1.1^+$	V males uninfected	V males infected	0.0035	F (3, 48) = 3.007
	T males uninfected	T males infected	0.009	F (3, 48) = 3.007
	LT males uninfected	LT males infected	0.0375	F (3, 48) = 3.007
	V males uninfected	V females uninfected	0.0003	F (6, 48) = 1.186
	V males infected	V females infected	0.045	F (6, 48) = 1.186
	T males infected	T females infected	0.0056	F (6, 48) = 1.186
	LT males uninfected	LT females uninfected	0.0187	F (6, 48) = 1.186
	LT males infected	LT females infected	0.0169	F (6, 48) = 1.186
4D. $CD3^+CD8^+$	V females uninfected	V females infected	0.0136	F (3, 48) = 3.393
	L females uninfected	L females infected	<0.0001	F (3, 48) = 3.393
	V males uninfected	V females uninfected	0.0219	F (6, 48) = 1.301
	L males uninfected	L females uninfected	0.0003	F (6, 48) = 1.301
	L males infected	L females infected	0.0067	F (6, 48) = 1.301
	T males uninfected	T females uninfected	0.0005	F (6, 48) = 1.301
	LT males uninfected	LT females uninfected	0.0206	F (6, 48) = 1.301
4E. IFN-γ	L females uninfected	L females infected	<0.0001	F (3, 60) = 45.54
	T females uninfected	T females infected	<0.0001	F (3, 60) = 45.54
	L males uninfected	L males infected	<0.0001	F (3, 60) = 45.54

	T males uninfected	T males infected	0.0009	F (3, 60) = 45.54
	L males infected	L females infected	<0.0001	F (9, 60) = 17.17
4F- IL-2	L females uninfected	L females infected	0.0012	F (3, 16) = 31.93
	T females uninfected	T females infected	<0.0001	F (3, 16) = 31.93
	L males infected	L females infected	0.0012	F (9, 45) = 15.93
	T males infected	T females infected	<0.0001	F (9, 45) = 15.93
5B. MAC3+	L females uninfected	L females infected	0.0007	F (3, 16) = 38.90
	T females uninfected	T females infected	0.0006	F (3, 16) = 38.90
	LT females uninfected	LT females infected	0.005	F (3, 16) = 38.90
	V males uninfected	V males infected	0.0206	F (9, 48) = 2.898
	L males uninfected	L males infected	0.0385	F (9, 48) = 2.898
	T males uninfected	T males infected	<0.0001	F (9, 48) = 2.898
	LT males uninfected	LT males infected	0.03	F (9, 48) = 2.898
	V males uninfected	V females uninfected	0.0015	F (9, 48) = 2.898
	L males uninfected	L females uninfected	<0.0001	F (9, 48) = 2.898
	T males uninfected	T females uninfected	<0.0001	F (9, 48) = 2.898
	LT males uninfected	LT females uninfected	<0.0001	F (9, 48) = 2.898
5C. IL-17A	V females uninfected	V females infected	<0.0001	F (3, 59) = 1.830
	L females uninfected	L females infected	<0.0001	F (3, 59) = 1.830
	T females uninfected	T females infected	<0.0001	F (3, 59) = 1.830
	LT females uninfected	LT females infected	<0.0001	F (3, 59) = 1.830
	V males uninfected	V females uninfected	<0.0001	F (9, 59) = 7.444
	L males uninfected	L females uninfected	<0.0001	F (9, 59) = 7.444
	L males infected	L females infected	0.0039	F (9, 59) = 7.444
	T males uninfected	T females uninfected	0.0003	F (9, 59) = 7.444
	LT males uninfected	LT females uninfected	<0.0001	F (9, 59) = 7.444
5D. TNF-α	L females uninfected	L females infected	<0.0001	F (3, 56) = 7.953
	T females uninfected	T females infected	<0.0001	F (3, 56) = 7.953
	L males infected	L females infected	0.0017	F (9, 56) = 4.684
	T males infected	T females infected	0.0011	F (9, 56) = 4.684
5E. IL-10	L females uninfected	L females infected	<0.0001	F (3, 60) = 45.54
	T females uninfected	T females infected	<0.0001	F (3, 60) = 45.54
	L males infected	L females infected	0.003	F (9, 60) = 17.17
6B. CD19+	V females uninfected	V females infected	<0.0001	F (3, 64) = 3.482
	L females uninfected	L females infected	<0.0001	F (3, 64) = 3.482
	V males uninfected	V males infected	0.0002	F (9, 64) = 7.126
	L males uninfected	L males infected	<0.0001	F (9, 64) = 7.126
	T males uninfected	T males infected	0.0005	F (9, 64) = 7.126
	LT males uninfected	LT males infected	0.0049	F (9, 64) = 7.126
	V males uninfected	V females uninfected	<0.0001	F (9, 64) = 7.126
	V males infected	V females infected	0.0009	F (9, 64) = 7.126
	L males uninfected	L females uninfected	<0.0001	F (9, 64) = 7.126
	T males uninfected	T females uninfected	<0.0001	F (9, 64) = 7.126
	LT males uninfected	LT females uninfected	<0.0001	F (9, 64) = 7.126
	LT males infected	LT females infected	0.0049	F (9, 64) = 7.126
6C. IL-4	L females uninfected	L females infected	0.0004	F (3, 42) = 5.946
	T females uninfected	T females infected	<0.0001	F (3, 42) = 5.946
	L males infected	L females infected	0.0223	F (9, 42) = 4.291
	T males infected	T females infected	<0.0001	F (9, 42) = 4.291
6D. IL-6	L females uninfected	L females infected	0.001	F (3, 16) = 15.10
	T females uninfected	T females infected	<0.0001	F (3, 16) = 15.10
	LT females uninfected	LT females infected	0.0023	F (3, 16) = 15.10
	L males infected	L females infected	0.001	F (9, 44) = 13.81

	T males infected	T females infected	<0.0001	F (9, 44) = 13.81
	LT males infected	LT females infected	0.0023	F (9, 44) = 13.81
6E. IgM	V males infected	V females infected	0.0036	F (9, 47) = 2.987
	LT males infected	LT females infected	0.0015	F (9, 47) = 2.987

DF = Degrees freedom, p-adj = adjusted p - values (p)
